# Whole-genome sequence-informed MALDI-TOF MS diagnostics reveal importance of *Klebsiella oxytoca* group in invasive infections: a retrospective clinical study

**DOI:** 10.1186/s13073-021-00960-5

**Published:** 2021-09-13

**Authors:** Aline Cuénod, Daniel Wüthrich, Helena M. B. Seth-Smith, Chantal Ott, Christian Gehringer, Frédéric Foucault, Roxanne Mouchet, Ali Kassim, Gunturu Revathi, Deborah R. Vogt, Stefanie von Felten, Stefano Bassetti, Sarah Tschudin-Sutter, Timm Hettich, Götz Schlotterbeck, Christina Homberger, Carlo Casanova, Jacob Moran-Gilad, Orli Sagi, Belén Rodríguez-Sánchez, Franco Müller, Martina Aerni, Valeria Gaia, Helke van Dessel, Greetje A. Kampinga, Claudia Müller, Claudia Daubenberger, Valentin Pflüger, Adrian Egli

**Affiliations:** 1 Applied Microbiology Research, Department of Biomedicine, University of Basel, Hebelstrasse 20, 4031 Basel, Switzerland; 2 Division of Clinical Bacteriology and Mycology, University Hospital Basel, Petersgraben 4, 4031 Basel, Switzerland; 3 Swiss Institute for Bioinformatics, Basel, Switzerland; 4 Division of Internal Medicine, University Hospital Basel, Basel, Switzerland; 5 Mabritec AG, Riehen, Switzerland; 6 Aga Khan University Hospital, Nairobi, Kenya; 7 Department of Clinical Research, University of Basel and University Hospital Basel, Basel, Switzerland; 8 Department of Biostatistics, Epidemiology, Biostatistics and Prevention Institute (EBPI), University of Zurich, Zurich, Switzerland; 9 Division of Infectious Diseases and Hospital Epidemiology, University Hospital Basel, Basel, Switzerland; 10 Department of Clinical Research, University of Basel, Basel, Switzerland; 11 Division of Instrumental Analytics, School of Applied Sciences (FHNW), Muttenz, Switzerland; 12 Institute for Infectious Diseases, University of Bern, Bern, Switzerland; 13 Faculty of Health Sciences, Ben-Gurion University of the Negev, Beer Sheva, Israel; 14 Soroka University Medical Center, Beer Sheva, Israel; 15 Hospital General Universitario Gregorio Marañón, Madrid, Spain; 16 Servicio de Microbiología Clínica y Enfermedades Infecciosas, Hospital General Universitario Gregorio Marañón, Instituto de Investigación Sanitaria Gregorio Marañón, Madrid, Spain. Instituto de Investigación Sanitaria Gregorio Marañón, Madrid, Spain; 17 Labor Team W AG, Goldach, Switzerland; 18 Servizio di microbiologia EOLAB, Ente Ospedaliero Cantonale, Bellinzona, Switzerland; 19 Department of Medical Microbiology, Maastricht University Medical Center, Maastricht, the Netherlands; 20 Department of Medical Microbiology & Infection prevention, University of Groningen, Groningen, the Netherlands; 21 University Medical Center Groningen (UMCG), Groningen, the Netherlands; 22 Laborgemeinschaft 1, Zurich, Switzerland; 23 Swiss Tropical and Public Health Institute, Basel, Switzerland; 24 Department of Sciences, University of Basel, Basel, Switzerland

**Keywords:** MALDI-TOF MS, *Klebsiella spp.*, Invasive infections, Antimicrobial resistance, Species identification

## Abstract

**Background:**

*Klebsiella* spp. are opportunistic pathogens which can cause severe infections, are often multi-drug resistant and are a common cause of hospital-acquired infections. Multiple new *Klebsiella* species have recently been described, yet their clinical impact and antibiotic resistance profiles are largely unknown. We aimed to explore *Klebsiella* group- and species-specific clinical impact, antimicrobial resistance (AMR) and virulence.

**Methods:**

We analysed whole-genome sequence data of a diverse selection of *Klebsiella* spp. isolates and identified resistance and virulence factors. Using the genomes of 3594 *Klebsiella* isolates, we predicted the masses of 56 ribosomal subunit proteins and identified species-specific marker masses. We then re-analysed over 22,000 Matrix-Assisted Laser Desorption Ionization - Time Of Flight (MALDI-TOF) mass spectra routinely acquired at eight healthcare institutions in four countries looking for these species-specific markers. Analyses of clinical and microbiological endpoints from a subset of 957 patients with infections from *Klebsiella* species were performed using generalized linear mixed-effects models.

**Results:**

Our comparative genomic analysis shows group- and species-specific trends in accessory genome composition. With the identified species-specific marker masses, eight *Klebsiella* species can be distinguished using MALDI-TOF MS. We identified *K. pneumoniae* (71.2%; *n* = 12,523), *K. quasipneumoniae* (3.3%; *n* = 575), *K. variicola* (9.8%; *n* = 1717), “*K. quasivariicola*” (0.3%; *n* = 52), *K. oxytoca* (8.2%; *n* = 1445), *K. michiganensis* (4.8%; *n* = 836), *K. grimontii* (2.4%; *n* = 425) and *K. huaxensis* (0.1%; *n* = 12). Isolates belonging to the *K. oxytoca* group, which includes the species *K. oxytoca*, *K. michiganensis* and *K. grimontii*, were less often resistant to 4th-generation cephalosporins than isolates of the *K. pneumoniae* group, which includes the species *K. pneumoniae*, *K. quasipneumoniae*, *K. variicola* and “*K. quasivariicola*” (odds ratio = 0.17, *p* < 0.001, 95% confidence interval [0.09,0.28]). Within the *K. pneumoniae* group, isolates identified as *K. pneumoniae* were more often resistant to 4th-generation cephalosporins than *K. variicola* isolates (odds ratio = 2.61, *p* = 0.003, 95% confidence interval [1.38,5.06])*. K. oxytoca* group isolates were found to be more likely associated with invasive infection to primary sterile sites than *K. pneumoniae* group isolates (odds ratio = 2.39, *p* = 0.0044, 95% confidence interval [1.05,5.53]).

**Conclusions:**

Currently misdiagnosed *Klebsiella* spp. can be distinguished using a ribosomal marker-based approach for MALDI-TOF MS. *Klebsiella* groups and species differed in AMR profiles, and in their association with invasive infection, highlighting the importance for species identification to enable effective treatment options.

**Supplementary Information:**

The online version contains supplementary material available at 10.1186/s13073-021-00960-5.

## Background

*Klebsiella* spp. are opportunistic pathogens, resident as respiratory and intestinal microbiota, and are commonly isolated during severe infections such as sepsis, pneumonia and pyelonephritis [1, 2]. Particularly hypervirulent strains of *K. pneumoniae*, which have been linked to specific capsular factors resulting in a muco-viscous phenotype, cause pyogenic liver abscesses and sepsis [3, 4]. In addition, the number of multi-drug resistant (MDR) isolates is increasing globally, carrying plasmids encoding for extended spectrum beta-lactamase (ESBL) or carbapenemase genes [5, 6]. *K. pneumoniae* is part of the ESKAPE pathogens (*Enterococcus faecium*, *Staphylococcus aureus*, *Klebsiella pneumoniae*, *Acinetobacter baumannii*, *Pseudomonas aeruginosa*, *Enterobacter*) which were classified by the WHO in 2017 as critical priority pathogens for research and development of new antibiotic treatment modalities [7].

**Fig. 1:**
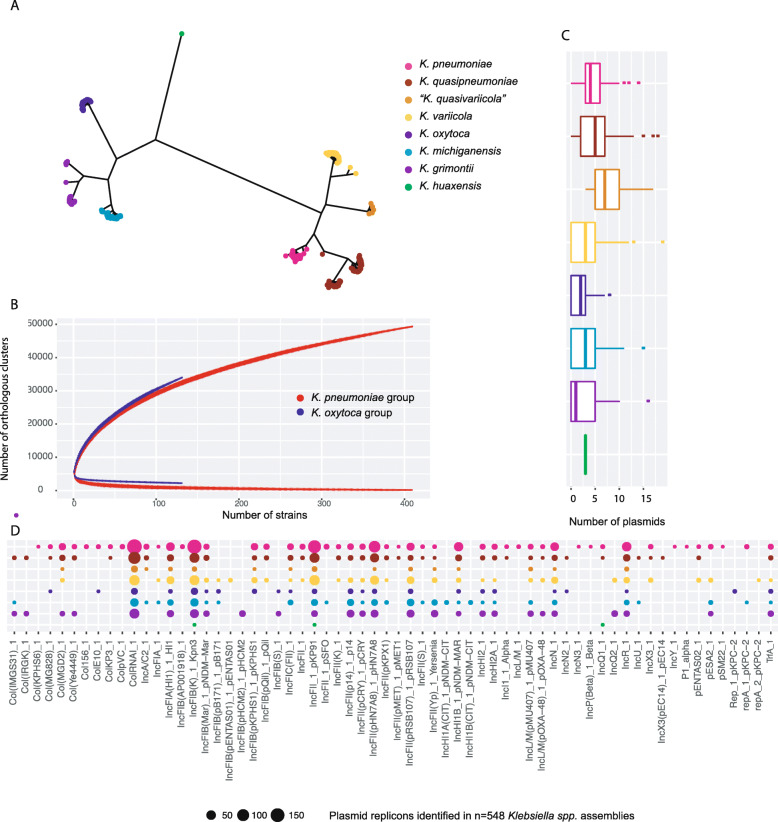
Genomic content of isolates across the *Klebsiella* genus. **A** Core-genome phylogeny of the genus *Klebsiella* including isolates from *K. pneumoniae* (*n =* 218), *K. quasipneumoniae* (*n =* 83), “*K. quasivariicola*” (*n =* 5), *K. variicola* (*n =* 109), *K. oxytoca* (*n =* 41), *K. michiganensis* (*n =* 54), *K. grimontii* (*n =* 37) and *K. huaxensis* (*n =* 1) based on 1171 core genes. The species colour key is used throughout the figure and paper. **B** Pan- (upper) and core- (lower) gene accumulative curves comparing *K. pneumoniae* group and *K. oxytoca* group. **C** Number of plasmid replicons identified in each isolate, per species. Boxes indicate the IQR with the median displayed as middle lines. **D** Plasmid replicons identified by PlasmidFinder in all isolates (*n =* 548), shown per *Klebsiella* spp

The taxonomy of the genus *Klebsiella* has been in flux for the past few years, divided into two main groups: the *K. pneumoniae* and the *K. oxytoca* group. Recently, several new species have been described within the *K. pneumoniae* group, which includes *K. pneumoniae* (sensu stricto), *K. quasipneumoniae* [8], *K. variicola* [9], “*K. quasivariicola*” [10] and *K. africana* [11]. The *K. oxytoca* group comprises *K. oxytoca* (sensu stricto), *K. michiganensis* [12], *K. grimontii* [13], *K. spallanzanii* and *K. pasteurii* [14]. *K. huaxensis* [15] is more closely related to the *K. oxytoca* group than to the *K. pneumoniae* group, but forms a distinct clade (Fig. 1A). Most of the *Klebsiella* spp. have been observed within a clinical context [10, 13–17]. The *K. oxytoca* group has been reported to cause antibiotic-associated hemorrhagic colitis in neonates, and hospital-acquired infections such as pneumonia and urinary tract infections (UTI) [18, 19]. Previous studies have analysed the population structure of a subset of *Klebsiella* spp. [10, 20, 21]. The gained knowledge from these studies could however not yet be translated to clinical routine diagnostics, and the clinical relevance of these recently described *Klebsiella* spp. is still unclear.

The newly described *Klebsiella* spp. are not yet identified with routine hospital-based diagnostic procedures. Biochemical reaction profiling cannot distinguish between all *Klebsiella* spp. [16, 22], neither is 16S rRNA a good sequencing target for species distinction within *Enterobacteriaceae* [23]. The most widely used technology for bacterial species identification in microbiological routine diagnostics is Matrix-Assisted Laser Desorption Ionization - Time Of Flight Mass Spectrometry (MALDI-TOF MS) [24]. The two commonly used commercial databases (MALDI Biotyper (MALDI Biotyper Compass Library, Revision E (Vers. 8.0, 7854 MSP, RUO) Bruker Daltonics, Bremen, Germany) and VitekMS DB (v.3.2, bioMérieux, Marcy-l’Étoile, France) allow spectral identification of *K. pneumoniae*, *K. variicola* and *K. oxytoca.* Importantly, “*K. quasivariicola*”, *K. quasipneumoniae*, *K. africana*, *K. michiganensis*, *K. grimontii*, *K. pasteurii*, *K. spallanzanii* and *K. huaxensis* are currently not included in these databases, and strains of these species are wrongly identified as either *K. pneumoniae* or *K. oxytoca* using MALDI-TOF MS [16]. Fortunately, recent developments show that a distinction of *Klebsiella* spp. is possible in routine diagnostics, using Fourier-transform infrared spectrometry [25] and MALDI-TOF MS using alternative databases [26].

Ribosomal subunit proteins are suitable as phylogenetic protein markers for MALDI-TOF mass spectra, as they are highly abundant in replicating cells and of relatively low molecular weight [27, 28]. Combinations of ribosomal subunit protein-derived masses allow the separation of sub-lineages within *Escherichia coli* [29] and *Streptococcus agalactiae* [30] by MALDI-TOF MS.

The aim of our study was to investigate the clinical presentation and distribution of AMR and virulence across the genus *Klebsiella*. Furthermore, using whole-genome sequences, we aimed to develop a ribosomal subunit-based MALDI-TOF MS scheme to robustly distinguish between *Klebsiella* spp. in clinical routine and to apply this on a large international dataset.

## Methods

An outline of the study method is given in Additional file 1: Figure S1.

### Ethics

Bacterial strains have been collected in clinical routine diagnostics. The collection of bacterial strains and their analysis for diagnostic assay development do not fall under the Swiss human research act, and no ethical approval nor consent to participate from patients was required. The analysis of patient demographic and clinical outcome data was approved by the “Ethikkommission Nordwest- und Zentralschweiz” (EKNZ) (BASEC-Nr. 2016-01899 and 2018-00225) for patients who did not reject the hospitals general research consent. Patients who did reject the hospital’s general consent were excluded from all analyses which include patient demographic and clinical outcome data.

### Bacterial isolates and whole-genome sequencing (WGS)

In total, 261 *Klebsiella* spp. isolates were collected from various tissue sources (see Additional file 2: Table S1 for more details) at three routine diagnostic laboratories in Switzerland including the University Hospital of Basel (USB; Basel, Switzerland), Mabritec AG (Riehen, Switzerland) and Labor Team W AG (LTW; Goldach, Switzerland)). Isolates were grown on Columbia 5% Sheep Blood Agar (bioMérieux, Marcy-l’Étoile, France), and DNA was extracted using the QIACube with the QIAamp DNA Mini Kit (QIAGEN, Hilden, Germany). After quality control of the DNA by Tapestation (Agilent, Santa Clara, USA), tagmentation libraries were generated as described by the manufacturer (Nextera XT kit, Illumina, San Diego, USA). The genomes were sequenced under 24× multiplexing using a 2 × 300 base pairs V3 reaction kit on an Illumina MiSeq instrument reaching an average coverage of approximately 60-fold for all isolates. Eleven isolates, covering reference and clinical isolates of 6 species were additionally sequenced on a PacBio Sequel at the Functional Genomics Center Zurich (FGCZ, ETH Zurich, Switzerland).

All available whole-genome assemblies designated as *Klebsiella* spp. were downloaded from NCBI in December 2017 (*n* = 3047), representing members of the species *K. pneumoniae*, *K. quasipneumoniae*, *K. variicola*, *“K. quasivariicola”*, *K. oxytoca*, *K. michiganensis*, *K. grimontii*, *K. huaxensis*, *K. aerogenes* and three species of the genus *Raoultella* (*R. ornithinolytica*, *R. planticola* and *R. terrigena*). An additional selection of publicly available *K. pneumoniae* whole-genome sequences were included from NCBI SRA, which was sampled to maximize diversity (*n =* 286) [20]. Two sets of *Klebsiella* spp. genomes were used for this study: first, a total of *n =* 3594 publicly available genome sequences including the 3333 described above, and the 261 sequenced at the USB, were used to in silico predict ribosomal protein masses. The species identity of these genome sequences were determined by comparison to the typestrains of *K. pneumoniae*, *K. quasipneumoniae*, *K. variicola*, “*K. quasivariicola*”, *K. oxytoca*, *K. michiganensis*, *K. grimontii*, *K. huaxensis R. ornithinolytica*, *R. planticola and R. terrigena* using Average Nucleotide Identity (ANIm) [31] and a threshold of 96%*.* Second, a computationally more manageable subset of these genomes (*n =* 999) was used for comparative genomic analyses, selected to represent the largest genomic diversity between and within species, and geographically. This subset included all assemblies of *K. quasipneumoniae*, *K. variicola*, “*K. quasivariicola*”, *K. oxytoca*, *K. michiganensis*, *K. grimontii* and *K. huaxensis*. For *K. pneumoniae*, only strains sequenced at USB and the previously published, diverse set of sequences [20] were included. To avoid bias introduced by outbreak strains, we excluded genomes which shared ANIm values > 99.9% with another genome in the collection, resulting in a final dataset of *n =* 548 genomes. Both datasets, including accession numbers and those of the short and long reads sequenced for this study, can be found in Additional file 2: Table S1. *K. africana*, *K. pasteurii* and *K. spallanzanii* were not included in this analysis as the species were not published at the time of the analysis and are extremely rare in clinics.

### Comparative genomic analysis

WGS data was quality controlled using FastQC [32] and MetaPhlAn (v2.0) [33] and adaptors were trimmed using Trimmomatic [34]. Genome assemblies were created using Unicycler (v0.4.4) [35]. Prokka (1.12) [36] was used for annotation. Orthologous groups were built using Roary (v3.10.2, option: -i 90) [37]. The resulting core-genome alignment was used for the construction of a phylogenetic tree using FastTree (v2.1) [38, 39]. The sizes of the core- and pan-genomes were calculated using a python script (https://github.com/appliedmicrobiologyresearch/Klebsiella-spp) [40].

The O-loci and K-loci were determined using KLEBORATE (v0.3.0) [41–43]. The genomes were investigated for the presence of known virulence loci (those included in KLEBORATE and the cytotoxin tilivalline [44]) and AMR determinants (via KLEBORATE). Potential plasmids were detected by comparing the genomes to the PlasmidFinder database [45] using abricate [46]. Genomic analyses were performed at sciCORE (http://scicore.unibas.ch/) scientific computing centre at University of Basel.

Scripts generating figures from the output of these tools were deposited on Github (https://github.com/appliedmicrobiologyresearch/Klebsiella-spp) [40].

### In silico prediction of ribosomal subunit protein masses from WGS data

The molecular weight of 56 ribosomal subunits was predicted in silico as described [26]: Tblastn (v 2.2.31+) was used to extract the amino acid sequences of 56 ribosomal subunits from 3594 *Klebsiella* spp. assemblies. Full ribosomal subunit sequences were retained when start and stop codons were identified and the length was within the median ± 3 codons. The subunits L1, L2 and S12 were not found in over 90% of the genomes and were therefore excluded from further analysis. The ribosomal subunit protein S1 was also excluded because S1-like domains are found in proteins unrelated to the ribosome [41]. The masses of the ribosomal subunit protein alleles were predicted including the N-end rule to account for possible methionine loss at the N-terminus [47]. The mass of subunit L33 was corrected by 15 Da to account for post translational methylation [48].

### Definition of species-specific MALDI-TOF MS marker masses

A diverse selection of bacterial isolates (*n =* 50) representing at least eight isolates of *K. pneumoniae*, *K. variicola*, *K. oxytoca*, *K. michiganensis* and *K. grimontii*, whole-genome sequenced for this study, were used to validate the detection of the predicted marker masses in MALDI-TOF mass spectra. These represent the most common species within the *K. pneumoniae* and the *K. oxytoca* group [5, 20, 21]. MALDI-TOF mass spectra of these 50 *Klebsiella* isolates were acquired on four MALDI-TOF MS systems in different laboratories, including one microflex Biotyper (Bruker Daltonics, Bremen, Germany) at the USB (Basel, Switzerland), one Axima Confidence (Shimadzu, Ngoyo, Japan) at Mabritec AG (Riehen, Switzerland) and two VitekMS devices (bioMérieux, Marcy-l’Étoile, France) at the Laborgemeinschaft 1 (LG1) (Zürich, Switzerland) and the Ente Ospedaliero Cantonale (Bellinzona, Switzerland). The *Klebsiella* isolates were measured on each system in quadruplicate using direct smear method and overlaid with formic acid (70% for the spectra acquired on the Microflex Biotyper and 25% for all other machines) and cyano-4-hydroxycinnamic acid (CHCA) matrix solution. MALDI-TOF mass spectra acquired on the VitekMS (bioMérieux, Marcy-l’Étoile, France) (*n =* 400 spectra) were output as .mzml files containing a list of peaks per spectrum. For MALDI-TOF mass spectra acquired on the Axima Confidence (*n =* 200 spectra) peak picking was performed using the Launchpad Software (v2.8, Shimadzu, Ngoyo, Japan) (parameters: scenario: “Advanced”; peak width: 80 chans; smoothing method: “Average”; smoothing filter width: 500 chans; peak detection method: “Threshold-Apex”; threshold type: “dynamic”; threshold offset: 0.025 mV; threshold response 1.25×). MALDI-TOF mass spectra acquired on a microflex Biotyper (*n =* 200 spectra) were output as fid-files and peak picking was performed in the flexAnalyses software (v3.4) (parameters: peak detection: “Centroid”; signal to noise threshold: 2; relative intensity threshold: 0%; minimal intensity threshold: 600; maximal number of peaks: 300; peak width: 4 m/z; peak height: 90%; baseline subtraction: “TopHat”; smoothing algorithm: “Savitzky Golay”; smoothing width: 2 m/z; smoothing cycles: 10). All MALDI-TOF mass spectra were internally calibrated with the conserved masses 4365.3 m/z, 6383.5 m/z, 7158.7 m/z, 7244.5 m/z, 10,286.1 m/z and a tolerance range of 1000 ppm, using the R-packages MALDIQuant and MALDIQuantForeign [49]. All spectra were exported in ASCII format and interrogated for the ribosomal subunit protein allele masses predicted from the respective WGS data. We used the following criteria to select the ribosomal target proteins for subsequent species identification, in order to maximize discriminatory power and reproducibility: ribosomal subunit protein masses in the mass range from 3000–13,000 Da (L36, S22, L34, L30, L32, L33, L35, L29, L31, S21, L27, S20, S15, S19, L25, S14, L21, L18) were selected if they were detected with a reproducibility > 80% in a least one centre, with the exception of ribosomal subunit L28, which had a maximal reproducibility of 57%. Additionally, ribosomal subunit protein masses in the higher mass range from 13,000 to 15,000 Da (L19, S13, L20, S8, L17, S9) were included, as they were detected with a reproducibility of at least 35% in at least one centre. Ribosomal subunits with a predicted molecular weight > 15,000 Da were not included for further analysis.

The bacterial species was assigned for which most marker masses could be detected in the acquired mass spectrum. If in a spectrum an equal number of marker masses from different *Klebsiella* species were found, the spectrum was assigned a multi-species ID (e.g. *K. michiganensis / K. oxytoca*) and labelled as “Multispecies ID only”.

### Classification of MALDI-TOF mass spectra acquired in routine microbiology diagnostics

Routinely acquired MALDI-TOF mass spectra (*n =* 33,160) from eight international healthcare institutions from four countries were analysed: the Soroka Medical Centre (SMC; Beer Sheva, Israel); the Hospital General Universitario Gregorio Marañón (HGUM; Madrid, Spain); the Servizio di microbiologia EOLAB, Ente Ospedaliero Cantonale (Bellinzona, Switzerland); the LG1, (Zurich, Switzerland); the LTW (Goldach, Switzerland); the USB (Basel, Switzerland); the Maastricht University Medical Center (MUM; Maastricht, the Netherlands) and the University Medical Center Groningen (UMCG; Groningen, the Netherlands). The MALDI-TOF mass spectra were processed as described above. Each routinely acquired spectrum was interrogated for the presence of the reproducibly detected ribosomal protein subunit derived mass combinations, with an accepted error range of 300 ppm. In total, 10,814 MALDI-TOF mass spectra were from duplicated bacterial isolates and excluded from further analysis, leading to a final number of 22,346 MALDI-TOF mass spectra representing unique bacterial isolates.

### Phenotypic profiling

The same collection of *Klebsiella* spp. strains (*n =* 50), which were used to define species-specific marker masses, were subjected to biochemical profiling on a Vitek2 (bioMérieux, Marcy-l’Étoile, France) using the GN ID card and the API50ch panel (bioMérieux, Marcy-l’Étoile, France). Primary metabolites of the same strains were measured and analysed as described in Additional file 3: Supplementary Methods. Additionally, 11 strains were subject to fatty acid profiling as described in Additional file 3: Supplementary Methods. These 11 strains included reference strains of the species *K. pneumoniae*, *K. quasipneumoniae*, *K. variicola*, *K. oxytoca* and *K. michiganensis*, one clinical isolate for each of the species *K. pneumoniae*, *K. variicola*, *K. oxytoca* and *K. michiganensis* and two clinical isolates of the species *K. grimontii.*

### Antimicrobial resistance determination

AMR profiles of isolates associated with the retrospectively analysed spectra were accessed through the laboratory information systems of the USB and the LTW (*n =* 7876). The accessed AMR profiles were measured in clinical routine diagnostics from January 2015 to June 2018 using either microdilution methods (Vitek2, AST-N242 GN Cards, bioMérieux), MIC strip tests (Liofilchem, Roseto degli Abruzzi, Italy) or disc diffusion tests (ThermoFisher Scientific, Waltham, USA). Breakpoints were interpreted as susceptible or resistant according to the current EUCAST Breakpoint table (v6.0 – 8.1) [50].

### Retrospective assessment and statistical analysis of clinical data

We assessed the relative distribution of *Klebsiella* spp. with regard to laboratory and country of isolation (*n =* 22,346, including spectra from eight laboratories). We examined the association of *Klebsiella* groups and species with resistance to antibiotic classes using logistic regression (post hoc analyses; *n =* 7876, including spectra from two laboratories).

Patient demographic and clinical data from patients with *Klebsiella* spp. infections were retrospectively accessed via the USB clinical information system in a case report form for a subset of clinical cases (*n =* 957). Inclusion criteria were as follows: patients for which at least one isolated bacterial colony was identified as *Klebsiella* spp. by MALDI-TOF MS collected between January 2015 and June 2018 at the USB and who did not reject the hospital’s general research consent form, as approved by the ethical committee. The USB is a tertiary healthcare centre with more than 750 beds in a low endemic region for ESBL-producing bacteria [51].

The clinical outcomes of 957 patients with *Klebsiella* infections were analysed and included all-cause mortality within 30 days from *Klebsiella* spp. diagnosis as primary endpoint and secondary endpoints: (i) time to death after *Klebsiella* spp. diagnosis in days, (ii) admission to an intensive care unit (ICU), (iii) invasive infection to sterile sites (including the bloodstream, deep tissues and cerebrospinal fluids), (iv) length of hospital stay in days, (v) the number of medical disciplines involved to manage the specific case (as a surrogate marker for case complexity) and (vi) whether the infection was mentioned in the patient letters. Clinical outcomes were examined for an association with distinct *Klebsiella* spp., age, sex, immunosuppression (defined as a dose equivalent of 20 mg prednisone / day or mentioning of immunosuppression in the patient notes), Charlson Comorbidity Index (CCI) [52], resistance to 3rd-generation cephalosporins and antibiotic treatment (defined as at least one dose of antimicrobial agent at hospital entry or during the hospital stay). Binary outcomes were analysed using generalized linear mixed models (GLMM) with binomial error distribution. Count outcomes (number of medical disciplines involved) were analysed using GLMM with Poisson error distribution. Time to death within hospital and length of hospital stay (time to discharge) were considered competing risks and jointly analysed by a competing risks model. For further detail, see Additional file 3: Supplementary Methods.

## Results

### Comparative genomic analyses of *Klebsiella* spp.

To determine the core-genome of the genus *Klebsiella*, from the 3594 *Klebsiella* spp. isolates with available WGS, a selection of 548 isolates was made, reflecting between- and within-species diversity. The genus core-genome comprises *n =* 1171 genes, which are genes shared between 99% of these 548 isolates. The core-genome-based phylogeny (Fig. 1A) clearly shows the *Klebsiella* phylogeny as previously described [5, 20, 21], with subspecies within *K. quasipneumoniae (K. quasipneumoniae* subsp. *quasipneumoniae* and *K. quasipneumoniae* subsp. *similipneumoniae*) as well as within *K. variicola* (*K. variicola* subsp. *variicola* and *K. variicola* subsp. *tropica*) which can be distinguished. These species also contain a diverse array of strains, in contrast to *K. pneumoniae*, which is more homogeneous when comparing these 1171 core genes. Interestingly, *K. grimontii* includes two deeply branching sub clades, which have not yet been described as subspecies (Fig. 1A).

The pan-genomes of the *K. pneumoniae* group and the *K. oxytoca* group were determined by investigating the number of unique orthologous clusters within our genome data set (Fig. 1B). A larger pan- to core-genome ratio could suggest adaptation to diverse environments, whereas a smaller pan- to core-genome ratio could, in a clinical context, reflect adaptation to the human host or even site-specific infections. Both groups show an open pan-genome, with the number of unique orthologous clusters increasing as more genomes are added to the analysis. There seems to be a larger increase in the pan-genome of the *K. oxytoca* group with strains added, although fewer genomes have been sampled to date. At a species level, pan-genome sizes of those within the *K. pneumoniae* group are relatively similar, whereas *K. michiganensis* within the *K. oxytoca* group shows a larger pan-genome, although this may reflect sampling bias (Additional file 1: Figure S2).

Plasmid complements are known to vary widely between *Klebsiella* isolates and can carry accessory genes involved in AMR and pathogenicity [53–55]. We detected a lower median number of plasmid replicons per isolate within the *K. oxytoca* group (median = 2, interquartile range (IQR) = 0–4) compared to isolates of the *K. pneumoniae* group (median = 4, IQR = 2-4) (Fig. 1C). The lowest median number of plasmids was detected for *K. grimontii* isolates (median = 1, IQR = 0–5), and the highest median number of plasmids detected in “*K. quasivariicola*” (median = 7, IQR = 5–10) isolates. We also observed that within the *K. pneumoniae* group, the median count of plasmids detected was lower in *K. variicola* isolates (median = 3, IQR = 0–5) than in isolates of the other species of the *K. pneumoniae* group (median = 4, IQR = 3–6 for *K. pneumoniae*; median = 5, IQR = 2–7 for *K. quasipneumoniae* and median = 7, IQR = 5–10 for “*K. quasivariicola*”) (Fig. 1C). No group specificity could be detected in plasmid replicon profiles (Fig. 1D). The two replicons known to be particularly related to virulence, plasmids KpVP-1 and KpVP-2, (IncHI1B_pNDM-MAR and IncFIB(K)_Kpn3 respectively), were detected in isolates from all *Klebsiella* spp. (*K. pneumoniae n =* 162/218, 73.3%; *K. quasipneumoniae n =* 61/83, 73.5%; *K. variicola n =* 56/109, 51.4%; “*K. quasivariicola*” *n =* 4/5, 80.0%; *K. oxytoca n =* 18/41, 43.9%; *K. michiganensis n =* 23/54, 42.6%; *K. grimontii n =* 16/37, 43.2%) with the exception of *K. huaxensis*, for which only a single genome was available for this study.

From long read genome assemblies for a subset of 11 isolates sequenced as part of this study, the plasmids assembled for nine isolates agreed with the replicon findings. In *K. quasipneumoniae* DSM-2811T, we detected a plasmid of 4267 bp with 99.1% identity and 71% coverage to the *K. pneumoniae* plasmid pB1019, which was not identified by PlasmidFinder. A previously undescribed plasmid of 3596 base pairs was identified within *K. grimontii* 606641-17, which does not carry any known virulence or resistance factors, showing that there is further diversity of *Klebsiella* spp. plasmids to discover.

#### Virulence factors of *Klebsiella* spp.

Virulence factors were investigated by comparing 548 *Klebsiella* spp. genomes against known databases and virulence factors. Genes encoding the iron-chelating siderophores aerobactin and salmochelin were detected in a minority of isolates (*n =* 18/548, 3.3% and *n =* 21/548, 3.8% of isolates, respectively), only within *K. pneumoniae* and *K. quasipneumoniae*, often co-occurring within isolates. The siderophore yersiniabactin is more prevalent in isolates of the *K. oxytoca* group (*n =* 111/132, 84.1%) than in the *K. pneumoniae* group (*n =* 52/415, 12.5%). The *kfu* operon, encoding an iron transport system, was detected in isolates of all species except *K. oxytoca* (*n =* 333/548; 60.8% of all isolates and *n =* 0/41; 0% within the species *K. oxytoca*)*.*

**Fig. 2:**
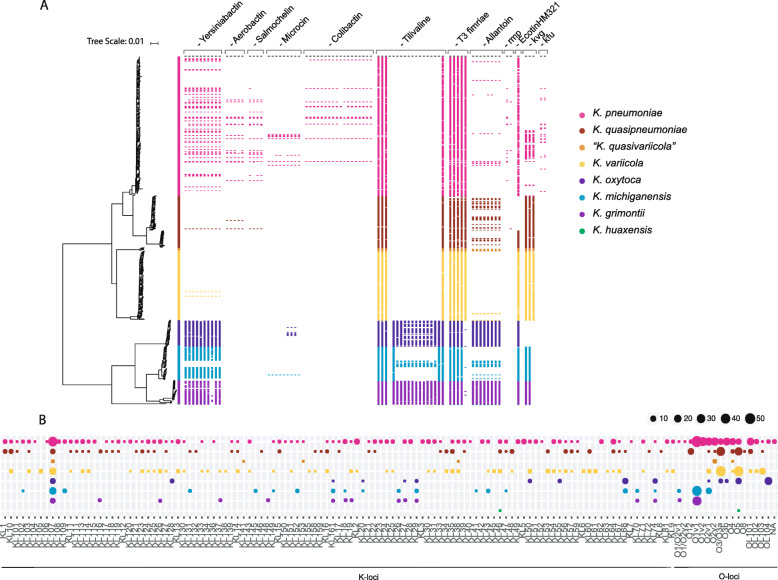
Virulence factors across the genus *Klebsiella*. **A** Core-genome phylogeny of 548 *Klebsiella* spp. genomes (left, in line with Fig. 1A) with identified virulence-related genes shown per isolate (right), coloured by species. **B** Polysaccharide (K-locus, left) and lipopolysaccharide (O-locus, right) predicted serotypes of isolates grouped by species

The genes involved in allantoin metabolism, which enable *Klebsiella* spp. to assimilate nitrogen from this metabolic intermediate and increase its virulence in certain infection sites, were detected in isolates of all species (*n =* 132/548; 24.1%), notably in all *K. oxytoca* (*n =* 41/41; 100%) and *K. grimontii* (*n =* 37/37; 100%) isolates*.* The distribution of bacterial toxin operons encoding microcin, colibactin and tilivalline was also examined. The complete microcin operon was detected in a few *K. pneumoniae* genomes (*n =* 8/218; 3.7%) and in one *K. michiganensis* genome (*n =* 1/54; 1.9%), whereas colibactin was detected solely in *K. pneumoniae* (*n =* 11/218; 5.0%)*.* In contrast, the complete tilivalline operon was exclusively identified in isolates within the *K. oxytoca* group (*n =* 64/132; 48.5%) particularly in isolates of *K. oxytoca* (*n =* 29/41; 59.2%) and *K. grimontii* (*n =* 30/37; 81.1%) (Fig. 2A).

The regulator of the mucoid phenotype *rmp* genes were detected in *K. pneumoniae* (*n =* 14/218; 6.4%) and *K. quasipneumoniae* (*n =* 1/83; 1.2%). Ecotin has been described as being able to modulate the host immune response [56] and was detected in isolates belonging to all species (*n =* 445/548; 81.2%).

We found a high diversity of K-loci in our dataset, with 114 capsule types. KL107 was found to be the most prevalent K-locus type across all *Klebsiella* spp. (107/548; 19.5%), with the exception of *K. huaxensis* where only one genome was included (KL46; Fig. 2B). KL1 and KL2, which are associated with a muco-viscous phenotype and frequently detected in hypervirulent strains, were exclusively detected in isolates of species within the *K. pneumoniae* group; KL1 was detected in isolates of *K. pneumoniae* (*n =* 4/218; 1.8%) and *K. quasipneumoniae* (*n =* 4/83; 4.8%), whereas KL2 was detected in isolates of *K. pneumoniae* (*n =* 10/218; 4.6%) and *K. variicola* (*n =* 1/109; 0.9%)*.* Within the O-loci, we found less diversity, with 17 types. Type O1v1 is the most common in isolates of *K. pneumoniae* (*n =* 53/218, 24.3%), *K. michiganensis* (*n =* 41/54; 75.9%) and *K. grimontii* (*n =* 36/37; 97.2%), whereas most isolates of *K. oxytoca* (*n =* 22/41; 53.7%) carry the O-locus OL104. The most common combination of K- and O-loci per species were as follows: KL107-O2v1, KL107-OL101 and KL64-O1v1 in *K. pneumoniae* (each 7/218; 3.2%), KL48-O5 in *K. quasipneumoniae* (*n =* 4/83; 4.8%) and KL107-O5 in *K. variicola* (*n =* 6/109; 5.5%). All five “*K. quasivariicola*” genomes studied carry unique combinations of K- and O-loci. Within the *K. oxytoca* group, the most common combination of K- and O-loci is carried by a bigger proportion of the isolates compared to the *K. pneumoniae* group species: KL68-O5 in *K. oxytoca* (*n =* 7/41; 17.1%), KL107-O1v1 in *K. michiganensis* (*n =* 11/54; 20.4%) and *K. grimontii* (*n =* 14/37; 41.2%) were the most frequently detected combinations.

#### Antimicrobial resistance genes in *Klebsiella* spp.

We examined the occurrence of AMR genes in *Klebsiella* spp*.* (*n =* 548) (Additional file 1: Figure S3). We detected the chromosomally encoded *AmpH* for strains of the *K. pneumoniae* group and a beta-lactamase of the *LEN* family in *K. variicola* isolates, which both confer low-level resistance to beta-lactam antibiotics [57]. Further, we detected the chromosomally encoded beta-lactamase genes *bla*_*OXY1*_*-bla*_*OXY8*_ in genomes within the *K. oxytoca* group, for which each species carries distinct variants, as described [58].

Fewer isolates of the *K. oxytoca* group were found to carry ESBL genes (8/132; 6.1%) than isolates of the *K. pneumoniae* group (125/415; 30.1%). Within the *K. pneumoniae* group, fewer isolates of the species *K. variicol*a were found to carry ESBL genes (14/109; 12.8%), compared to isolates within *K. pneumoniae* (78/218; 35.8%), *K. quasipneumoniae* (32/83; 38.6%) and “*K. quasivariicola*” (1/5; 20.0%). We observed a higher number of *K. quasipneumoniae* isolates encoding carbapenemases (12/83; 14.4%) compared to *K. pneumoniae* (15/218; 6.9%), *K. variicola* (9/109; 8.6%) and “*K. quasivariicola*” (0/5; 0%). Within the *K. oxytoca* group, we detected the highest frequency of ESBL and carbapenemases in *K. michiganensis* (6/54; 11.1% and 5/54; 9.3%, respectively) compared to *K. oxytoca* (both in 1/41; 2.4%) and *K. grimontii* (both in 2/37; 5.4%).

### *Klebsiella* spp. identification in routine diagnostics

Given the group- and species-specific trends in accessory genome composition and content, an accurate species identification may have an important clinical impact. For the strains included in this study, fatty acid analysis, GC-MS and a panel of biochemical reactions were unable to identify a characteristic feature that could be used to distinguish unambiguously between the included *Klebsiella* spp. (Additional file 4: Table S2, Additional file 1: Figure S4, Additional file 5: Table S3). As such, in order to find a robust and accurate way to distinguish *Klebsiella* spp. based on MALDI-TOF mass spectra, we used ribosomal subunit proteins as species-specific MALDI-TOF MS markers. To do this, we first in silico-predicted protein masses of the 56 ribosomal subunit proteins from 3594 genome drafts.

Only proteins with a mass between 2000 and 20,000 Da can be detected in MALDI-TOF mass spectra, and due to the intrinsic measurement error of MALDI-TOF MS, not all predicted masses can be distinguished. Additionally, not all ribosomal subunit proteins can be equally ionized, and therefore detected, in similar proportions. Therefore, to determine the practical value of our approach, we examined which of these potential marker masses can reproducibly be detected in MALDI-TOF mass spectra of routine quality. Fifty isolates, representing the species *K. pneumoniae* (*n =* 10), *K. variicola* (*n =* 10), *K. oxytoca* (*n =* 8), *K. michiganensis* (*n =* 12) and *K. grimontii* (*n =* 10), all with species identification confirmed by WGS, were analysed in quadruplicate on four different MALDI-TOF MS systems in different laboratories, resulting in the generation of 800 spectra. The 25 ribosomal subunits L17, L18, L19, L20, L21, L25, L27, L28, L29, L30, L31, L32, L33 (methylated), L34, L35, L36, S8, S9, S14, S13, S15, S19, S20, S21 and S22 were subsequently included as valid target proteins for identification of *Klebsiella* spp. The reproducibility of these marker masses varied between the different laboratories (Additional file 6: Table S4). We found a complete set of these 25 target proteins in 3360 assemblies and therefore based our further analyses on these predicted mass profiles (Additional file 7: Table S5).

**Fig. 3:**
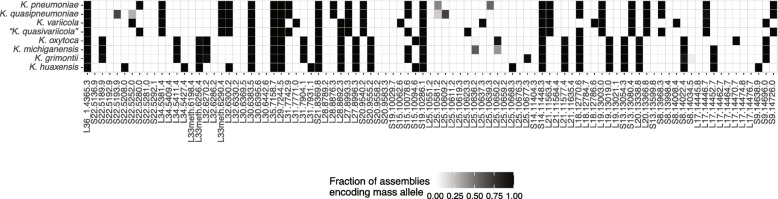
Distribution of the in silico-predicted masses of 25 target proteins encoded by the species *K. pneumoniae* (*n =* 208), *K. quasipneumoniae* (*n =* 60), *K. variicola* (*n =* 68), “*K. quasivariicola*” (*n =* 3), *K. oxytoca* (*n =* 37), *K. michiganensis* (*n =* 51), *K. grimontii* (*n =* 36) and *K. huaxensis* (*n =* 1)

We assessed the distribution of the in silico*-*predicted masses of these target proteins in a representative dataset of 464 genomes, comprising those which were included in the comparative genomic analysis and were complete for these 25 target proteins. We identified multiple group- and species-specific alleles for *K. pneumoniae*, *K. variicola*, “*K. quasivariicola*”, *K. oxytoca* and *K. grimontii* (Fig. 3). No marker mass uniquely identifying *K. michiganensis* and *K. quasipneumoniae* was detected. In order to unambiguously separate *K. michiganensis* and *K. quasipneumoniae* from closely related species, a combination of marker masses needs to be detected (e.g. S15 at 10,078 Da and L25 at 10,636 Da for *K. michiganensis*, Fig. 3).

Based on the differential masses of the 25 target proteins, we can distinguish 110 distinct *Klebsiella* spp. ribosomal mass profiles (Additional file 8: Table S6) that allow us to distinguish *K. pneumoniae*, *K. quasipneumoniae*, *K. variicola*, *"K. quasivariicola"*, *K. oxytoca*, *K. michiganensis*, *K. grimontii* and *K. huaxensis*. These 110 distinct ribosomal mass profiles were subsequently included in an in house developed reference database for species identification. We assessed the specificity and sensitivity of this approach, using the spectra (*n =* 800) of the same diverse *Klebsiella* spp. isolates (*n =* 50). The species identification by MALDI-TOF MS was compared to the species identity as assigned by WGS of the identical isolate. If essential target proteins could not be detected in a MALDI-TOF mass spectrum and the acquired mass profile did not allow unique species identification, this spectrum was labelled as “Multispecies ID only”. Table 1 shows the evaluation of the species identification of these spectra, resulting from comparison of the acquired MALDI-TOF mass spectra with the 110 ribosomal marker mass profiles. The identification was evaluated on two levels: (i) whether the assignment to *K. pneumoniae* or *K. oxytoca* group was correct and (ii) whether the correct species within each group could be assigned. Species identification using these marker mass profiles resulted generally in accurate identification and provided better species identification within the *K. oxytoca* group than the currently used commercially available databases Microflex Biotyper Database (MALDI Biotyper Compass Library,Revision E (Vers. 8.0, 7854 MSP, RUO) ) and the VitekMS Database (v.3), as these databases do not include *K. grimontii* and *K. michiganensis*.

**Table 1:** Evaluation of the species identification of 50 *Klebsiella* spp. isolates by MALDI-TOF mass spectra using ribosomal marker mass profiles. Species identification of 800 MALDI-TOF mass spectra using 110 marker mass profiles was compared to the species identification assigned using WGS data. Specificity and sensitivity were computed on two levels: (i) whether the assignment to *K. pneumoniae* group or *K. oxytoca* group was correct (= “Group level”) and (ii) whether the correct species within each group could be assigned (= “Species level”)

Species identification by WGS	Identification of MALDI-TOF mass spectra using Marker Mass profiles based on 25 pre-defined ribosomal subunit proteins			
	Group level		Species level	
	Sensitivity [%]	Specificity [%]	Sensitivity [%]	Specificity [%]
*K. pneumoniae*	98.8	100	70.0	100
*K. variicola*	98.1	100	93.1	100
*K. oxytoca*	99.2	100	80.5	99.4
*K. michiganensis*	100	100	44.3	100
*K. grimontii*	99.4	100	70.6	100

The sensitivity and specificity of the approach (Table 1) were computed based on the identification of MALDI-TOF mass spectra acquired on all four MALDI-TOF MS systems. Specificity and sensitivity on group level were > 98% using marker mass profiles, for all tested species on all four MALDI-TOF MS systems, reflecting a low probability of false positive results using this approach.

Sensitivity on the species level varied between the MALDI-TOF MS systems, especially within the *K. oxytoca* group. For the species *K. oxytoca*, sensitivity at the species level ranged from 37.5 to 100% between the four MALDI-TOF MS systems, for *K. michiganensis* from 8.3 to 70.8% and for *K. grimontii* from 12.5 to 95.0%. Species identification within the *K. oxytoca* group requires the detection of marker masses in a high mass range and variations in sensitivity between the MALDI-TOF MS systems could potentially be linked to MALDI-TOF mass spectral quality [59].

### Classification of international routinely acquired MALDI-TOF mass spectra

**Fig. 4:**
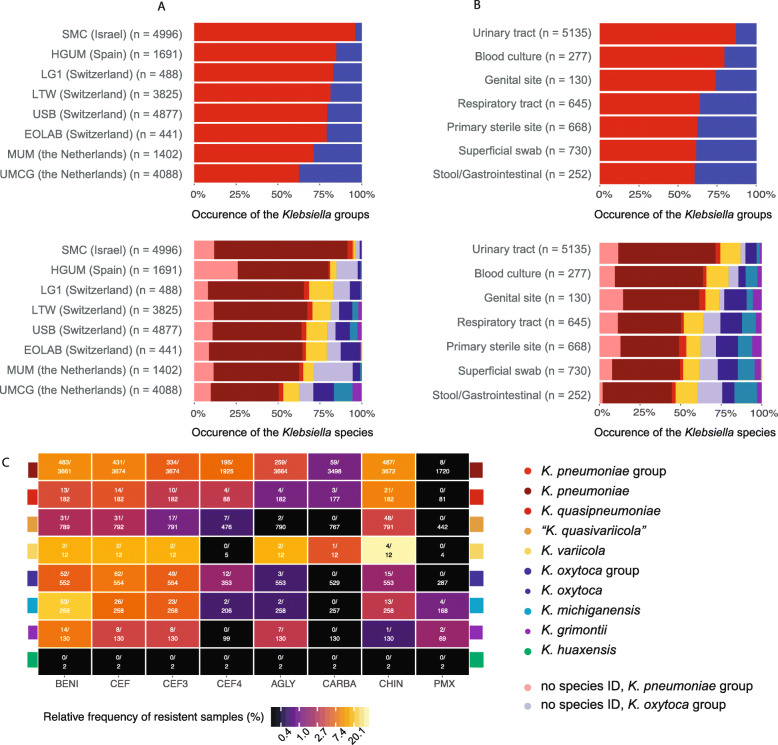
Occurrence of *Klebsiella* spp. in clinical settings, as determined by ribosomal marker MALDI-TOF MS method. **A** Occurrence of *Klebsiella* groups and species in eight healthcare centres from Israel (*n =* 1), Spain (*n =* 1), Switzerland (*n =* 4) and the Netherlands (*n =* 2), sorted by increasing occurrence of the *K. oxytoca* group. **B** Occurrence of *Klebsiella* groups and species in patient samples from various isolation sites. “Primary sterile sites” includes deep wounds, aspirates and deep tissues; “Respiratory tract” includes sputum, bronchoalveolar lavage and tracheal secretion; “Superficial swabs” includes swabs from superficial wounds and skin infections. **C** Antibiotic resistance of *Klebsiella* spp. (BENI = beta lactams with beta-lactamase inhibitors, CEF3 = 3rd-generation cephalosporins, CEF4 = 4th-generation cephalosporins, AGLY = aminoglycosides, CARBA = carbapenems, CHIN = quinolones, PMX = polymyxins). Please note that the colour grading is on log-scale

Using the 110 species-specific marker mass profiles, we retrospectively analysed 22,346 spectra derived from bacterial isolates from eight healthcare centres in four countries (dataset (a), Additional file 1: Figures S1 and S5). All spectra had previously been used to diagnose isolates as *Klebsiella* spp. in routine diagnostic laboratories. Re-analysing the spectra using the newly compiled mass profile database, we attempted to categorize all spectra first into the two groups and then to one of the eight species in the database (Fig. 4A, B).

A subset of the samples (*n =* 427; 1.9%) were identified as *Raoultella* spp. or *K. aerogenes* and excluded from further analysis. In total, 85 samples (0.3%) could only be identified to the genus level. The remaining 21,834 samples (97.7%) were categorized as *K. pneumoniae* group or *K. oxytoca* group. Of these, a higher proportion of samples could be categorized to the species level within the *K. pneumoniae* group (*n =* 14,867/17,555; 84.7%), than within the *K. oxytoca* group (*n =* 2718/4249; 64.0%), which reflects the difficulty to reproducibly detect all required species-specific marker masses in the *K. oxytoca* group. The proportion of samples which could not be categorized to the species level varied by healthcare centre and MALDI-TOF MS system and ranged from 9.8% (*n =* 40/407 samples) to 30.5% (*n =* 439/1440 samples) within the *K. pneumoniae* group and from 22.8% (*n =* 349/2560 samples) to 84.8% (*n =* 217/256 samples) within the *K. oxytoca* group. The remaining 17,585 samples (78.69%) could unambiguously be identified to the species level. Of these, across all centres, we identified: *K. pneumoniae* (*n =* 12,523; 71.2%), *K. quasipneumoniae* (*n =* 575; 3.3%) *K. variicola* (*n =* 1717; 9.8%), “*K. quasivariicola*” (*n =* 52; 0.3%), *K. oxytoca* (*n =* 1445; 8.2%), *K. michiganensis* (*n =* 836; 4.8%), *K. grimontii* (*n =* 425; 2.4%) and *K. huaxensis* (*n =* 12; 0.1%).

Interestingly, we observed different frequencies of the two *Klebsiella* groups and species depending on the originating healthcare centre, possibly reflecting a geographical trend of pathogenic *Klebsiella* spp. distribution (Fig. 4A): The proportion of isolates belonging to the *K. oxytoca* group was higher in more northern regions, a finding which requires further investigation. The proportion of *Klebsiella* groups and species was also found to vary depending on the patient material from which it was isolated (Fig. 4B). Isolates of the *K. oxytoca* group were least abundant in urinary tract samples and most abundant in samples of the gastro-intestinal tract samples.

### AMR profiles

Information on phenotypic AMR and isolation source was available for 7876 samples from two healthcare centres in Switzerland (USB and LTW), (dataset (b), Fig. 4C, Additional file 1: Figures S1 and S5) for which spectra had been analysed. Isolates of the *K. oxytoca* group were more likely to be resistant against penicillins including beta-lactamase inhibitors and 3rd-generation cephalosporins (OR 2.79, *p* < 0.001, 95% CI [1.70, 4.63]; OR 2.45, *p* = 0.005, 95% CI [1.31, 4.58], respectively) but less often resistant to 4th-generation cephalosporins and aminoglycosides (OR 0.17, *p* < 0.001, 95% CI [0.09, 0.28]; OR 0.22, *p* < 0.001, 95% CI [0.12, 0.35]) than isolates of the *K. pneumoniae* group (Additional file 9: Tables S7 - S9). Within the *K. oxytoca* group we found *K. oxytoca* to be less resistant to penicillins including beta-lactamase inhibitors, than *K. michiganensis* (OR 0.57, *p* < 0.001, 95% CI [0.42, 0.75]) (Additional file 9: Table S7). Within the *K. pneumoniae* group, isolates identified as *K. pneumoniae* were more resistant to penicillins including beta-lactamase inhibitors, 3rd- and 4th-generation cephalosporins as well as to aminoglycosides than *K. variicola* (OR 2.11, *p* < 0.001, 95% CI [1.42, 3.18]; OR 2.61, *p* < 0.003, 95% CI [1.38,5.06] and OR 5.80, *p* < 0.001, 95% CI [2.40,20.04], respectively) (Additional file 9: Tables S8-S10).

### Clinical endpoints

Data from patient charts of 957 clinical cases at the USB were reviewed and analysed on multiple clinical endpoints (dataset (c), Additional file 1: Figures S1 and S5). In order to examine the clinical phenotype of the *Klebsiella* groups and species, independent of their AMR burden, we corrected our model for resistance against 3rd-generation cephalosporins, which is associated with production of ESBL. Clinical outcomes and explanatory variables are summarized in Additional file 10: Tables S11-S12*.*

We found no evidence for *Klebsiella* group- or species-specific association with our primary 30-day mortality endpoint (Additional file 10: Table S13). As a general finding, female patients seemed to have better outcomes than male patients: all-cause 30-day mortality was less likely for female patients (OR 0.60, *p* = 0.012, 95% CI [0.40, 0.89]), female patients were less likely to be affected by invasive infection of sterile sites (OR 0.54, *p* = 0.002, 95 % CI [0.37, 0.79]) and to be admitted to an ICU (OR 0.63, *p* = 0.009, 95 % CI [0.45, 0.89]) (Table 2, Additional file 10: Tables S13 - S14). Furthermore, increasing CCI and increasing age seemed to be associated with higher 30-day mortality (OR 1.16, *p* = 0.40, 95% CI [1.01,1.34], and OR 1.36, *p* < 0.001, 95% CI [1.24,1.49], respectively), whereas antibiotic treatment at entry or during hospitalization was associated with higher odds for ICU admission (OR 4.41, *p* < 0.001 and 95% CI [2.10,8.93]) (Tables S15 – S16).

**Table 2:** Odds ratio estimates for invasive infection using the generalized linear mixed-effects model (GLMM). *n =* 732 complete observations with 162 events. OR = odds ratio; CI = confidence interval; CCI = Charlson Comorbidity Index

	OR	95 % CI	***Z***	***p*** value
***K. oxytoca*group*vs. K. pneumoniae*group**	2.39	[1.05,5.53]	2.01	0.044
***K. oxytoca*vs.*K. michiganensis***	0.75	[0.42,1.34]	− 0.97	0.33
***K. oxytoca vs. K. grimontii***	0.64	[0.30,1.36]	− 1.15	0.252
***K. pneumoniae vs. K. variicola***	1.08	[0.69,1.65]	0.35	0.724
***K. pneumoniae vs. K. quasipneumoniae***	0.64	[0.35,1.15]	− 1.44	0.149
**CCI**	1.14	[1.04,1.25]	2.95	0.003
**Age (centred, 10 years increase)**	0.84	[0.75,0.95]	− 2.78	0.005
**Female (ratio)**	0.54	[0.37,0.79]	− 3.17	0.002
**Immunosuppression**	0.5	[0.29,0.86]	− 2.5	0.012
**Resistance to 3rd-generation cephalosporins**	1.31	[0.44,3.74]	0.5	0.618
**Antibiotic treatment at entry or during hospitalization**	3.11	[1.44,6.49]	2.92	0.003

Strikingly, isolates of the *K. oxytoca* group were more likely to be involved in invasive infection compared to isolates of the *K. pneumoniae* group (OR 2.39, *p* = 0.044, 95% CI [1.05,5.53]). As we corrected in our model for resistance against 3rd-generation cephalosporins, and as the *K. oxytoca* group is not associated with a higher burden of AMR, we hypothesize that this increased association to invasive infections is independent of AMR and might reflect increased virulence of this group.

We found no evidence for *Klebsiella* group- or species-specific associations with the remaining clinical outcomes (Additional file 10: Tables S14 – S17).

## Discussion

We have described a MALDI-TOF MS method allowing the identification of clinically important and currently often misdiagnosed *Klebsiella* spp. and applied it to an international dataset of over 22,000 unique bacterial isolates from microbiological routine laboratories. While species-specific MALDI-TOF MS patterns within the genus *Klebsiella* have previously been described [8, 25, 60], their discriminatory power has not yet been assessed in large routinely acquired mass spectral datasets.

Using our ribosomal marker-based approach, we are able to separate eight species of the genus *Klebsiella*: *K. pneumoniae*, *K. quasipneumoniae*, *K. variicola*, “*K. quasivariicola*”, *K. oxytoca*, *K. michiganensis*, *K. grimontii* and *K. huaxensis*. This higher phylogenetic resolution power represents an important step forward in clinical diagnostics as “*K. quasivariicola*”, *K. michiganensis*, *K. grimontii* and *K. huaxensis* are currently not found in commonly used databases. Mass spectral quality plays an important role in distinguishing the species within the *K. oxytoca* group, as the species-specific peaks lie in a high mass range with m/z values above 10,000. Moreover, in order to unambiguously identify the species *K. michiganensis*, a unique combination of marker masses in a higher mass range needs to be detected, posing an additional challenge for identification. The inability to detect these in many spectra decreases sensitivity. We evaluated our approach by computing sensitivity and specificity values for five clinically important *Klebsiella* spp.. A limitation of the current study is that the most recently described *Klebsiella* spp., *K. africana*, *K. pasteurii* and *K. spallanzanii* were not included in the analysis. Identifying species-specific marker masses for these, and a similar evaluation for the less frequently observed *Klebsiella* species would be desirable in future. A recent study introduced a web-based tool which uses other core proteins than ribosomal subunit proteins to distinguish between the *Klebsiella* spp. [26]. Combining these and our ribosomal marker masses could potentially increase the resolution of MALDI-TOF mass spectral identification, even below the species level.

Marker-based approaches are independent of the MALDI-TOF MS system used. Therefore, we were able to assess the occurrence and clinical phenotype of important *Klebsiella* spp. in international clinical laboratories using MALDI-TOF MS systems from different manufacturers. While *K. pneumoniae* remains the most commonly detected species, we also detected isolates from each of the species, which are currently not routinely identified by common MALDI-TOF MS databases, including *K. quasipneumoniae*, “*K. quasivariicola*”, *K. michiganensis*, *K. grimontii* and *K. huaxensis* from a variety of patient material.

Our data provide evidence that infections with isolates of the *K. oxytoca* group were more likely to be invasive than infections with isolates of the *K. pneumonia* group, highlighting the clinical importance of this under-appreciated group. The clinical cases analysed in this study were treated at the same healthcare centre in a low endemic region for ESBL-producing bacteria. Further studies from different regions would be needed in order to confirm these results in other epidemiological backgrounds.

Due to the higher prevalence of the *K. pneumoniae* group infections, a larger absolute number of invasive infections are caused by isolates of the *K. pneumoniae* group than by isolates of the *K. oxytoca* group. Thus, infections with a *K. oxytoca* group isolate tend to be less frequent, but more severe, a finding which could potentially be linked to the increased frequency of certain virulence factors, such as the siderophore yersiniabactin, genes involved in allantoin metabolism and the cytotoxin tilivallin. Yersiniabactin and genes involved in the allantoin metabolism are well established virulence factors of *K. pneumoniae* [61, 62], and their frequent occurrence in assemblies of the *K. oxytoca* group has been observed before [21, 63]. Based on the data acquired in this study, we describe an indirect link between the association of *K. oxytoca* group strains and invasive infection and the occurrence of these virulence factors in genomes of the same species. However, in order to assess whether these factors actually increase the virulence of *K. oxytoca* group strains, functional studies are needed.

Bloodstream infections caused by *K. variicola* have been described as causing a higher mortality than bloodstream infections caused by *K. pneumoniae* [64], a finding that we could not confirm in our dataset.

Resistance to 3rd-generation cephalosporins is most often conferred by ESBLs, which were enriched in the analysed genomes of *K. pneumoniae* group compared to the *K. oxytoca* group (Additional file 1: Figure S3). Within the *K. pneumoniae* group isolates for which spectra were analysed, we observed a higher proportion of isolates resistant to 3rd- and 4th-generation cephalosporins and aminoglycosides in *K. pneumoniae* compared to *K. variicola*. The lower proportion of resistant isolates within *K. variicola* has previously been described [65] and is also in line with the lower frequency of ESBL genes detected in *K. variicola* genome sequences, compared to *K. pneumoniae* genome sequences. As genes encoding AMR are often carried on plasmids, this may be linked to the lower median number of plasmids detected in isolates of *K. variicola.*

These findings need to be explored in other epidemiological situations with higher ESBL and carbapenemase burdens to examine their broader relevance. With this, a rapid and accurate species identification by MALDI-TOF MS may have an important impact on antibiotic stewardship and treatment decisions.

## Conclusions

Based on systematic comparison of WGS and in silico ribosomal subunit protein mass prediction, we present a MALDI-TOF MS-based analytical approach to distinguish eight *Klebsiella* species which can be applied in clinical routine diagnostic laboratories. In this study, we identified species-specific AMR and virulence patterns within the genus *Klebsiella* and uncovered an increased association of the *K. oxytoca* group with invasive infection to primary sterile sites.

## Supplementary Information

**Additional file 1: Figure S1.** Schematic representation of the workflow of the project. **Figure S2.** Gene accumulation curves for species of the *K. pneumoniae* group (A) and the *K. oxytoca* group (B). **Figure S3.** Genes associated with AMR detected in *Klebsiella spp.***Figure S4.** Partial least squares discriminant analysis (PLS-DA) score plot containing primary metabolites measured of five *Klebsiella spp.***Figure S5.** Species identity of datasets (a), (b) and (c) included in the statistical analysis.

**Additional file 2: Table S1.** Strains included in the study and where sequence data can be accessed.

**Additional file 3.** Supplementary methods for: Measurement of primary metabolites. Fatty acid analysis. Statistical analysis of clinical outcome data.

**Additional file 4: Table S2.** Cellular fatty acid composition of 11 *Klebsiella* spp. strains.

**Additional file 5: Table S3.** Biochemical reaction and AMR profiles of a diverse set of *Klebsiella* spp. Strains.

**Additional file 6: Table S4.** Reproducibility of detection for the predicted ribosomal subunits in MALDI-TOF mass spectra acquired in 4 different centers.

**Additional file 7: Table S5.** Binary table displaying the predicted ribosomal subunits mass variants and whether this variant was predicted from an assembly or not.

**Additional file 8: Table S6.** Binary table of the protein marker masses which can reproducibly be detected in MALDI-TOF MS spectra.

**Additional file 9: **Odds ratio estimates comparing *Klebsiella* groups and species for resistance to different antibiotic classes: **Table S7.** Penicillins with beta-lactamase Inhibitors. **Table S8.** 3rd-generation cephalosporins. **Table S9.** 4th-generation cephalosporins. **Table S10.** Aminoglycosides.

**Additional file 10: **Summary data compiled for the statistical analysis of clinical endpoints: **Table S11.** Summary of outcome variables for the clinical data set. **Table S12.** Summary of explanatory variables for the clinical data set.
Statistical analyses examining differences in clinical outcome between the *Klebsiella* groups and species: **Table S13.** Odds ratio estimates from the generalized linear mixed-effects model (GLMM) for all cause death within 30 days from diagnosis. **Table S14.** Odds ratio estimates from the generalized linear mixed-effects model (GLMM) for ICU admission. **Table S15.** Hazard ratio estimates from cause-specific hazards Cox proportional hazards model for time to death within hospital after diagnosis with hospital discharge as competing event. **Table S16.** Estimates of the multiplicative effects from the Poisson generalized linear mixed-effects model (GLMM) for the number of medical disciplines involved. **Table S17.** Odds ratio estimates from the generalized linear mixed-effects model (GLMM) for the mentioning of the infection in the patient letter.

## Data Availability

Whole-genome sequences acquired for this study have been uploaded to Genebank (https://www.ncbi.nlm.nih.gov/genbank/). Accession numbers of these and previously published WGS data used in this study can be found in Additional file 2: Table S1. Software code generating figures from the genomic analysis is available on GitHub (https://github.com/appliedmicrobiologyresearch/Klebsiella-spp) [40].

## Abbreviations

AMR: Antimicrobial resistance
WGS: Whole-genome sequencing
MDR: Multi-drug resistant
MALDI-TOF MS: Matrix-Assisted Laser Desorption Ionization - Time Of Flight Mass Spectrometry
ANI: Average Nucleotide Identity
CHCA: Cyano-4-hydroxycinnamic acid
ICU: Intensive care unit
CCI: Charlson Comorbidity Index
GLMM: Generalized linear mixed models
IQR: Interquartile range
OR: Odds ratio
