# Supplementary material for: Whole-genome sequence-informed MALDI-TOF MS diagnostics reveal importance of Klebsiella oxytoca group in invasive infections: a retrospective clinical study
Source: Genome Med. 2021 Sep 13;13:150. doi: 10.1186/s13073-021-00960-5 (PMC8438989; doi:10.1186/s13073-021-00960-5)
Supplement: Supplementary file 1 — Additional file 1: Figure S1. Schematic representation of the workflow of the project. Figure S2. Gene accumulation curves for species of the K. pneumoniae group (A) and the K. oxytoca group (B). Figure S3. Genes associated with AMR detected in Klebsiella spp.Figure S4. Partial least squares discriminant analysis (PLS-DA) score plot containing primary metabolites measured of five Klebsiella spp.Figure S5. Species identity of datasets (a), (b) and (c) included in the statistical analysis. [file 13073_2021_960_MOESM1_ESM.pdf]

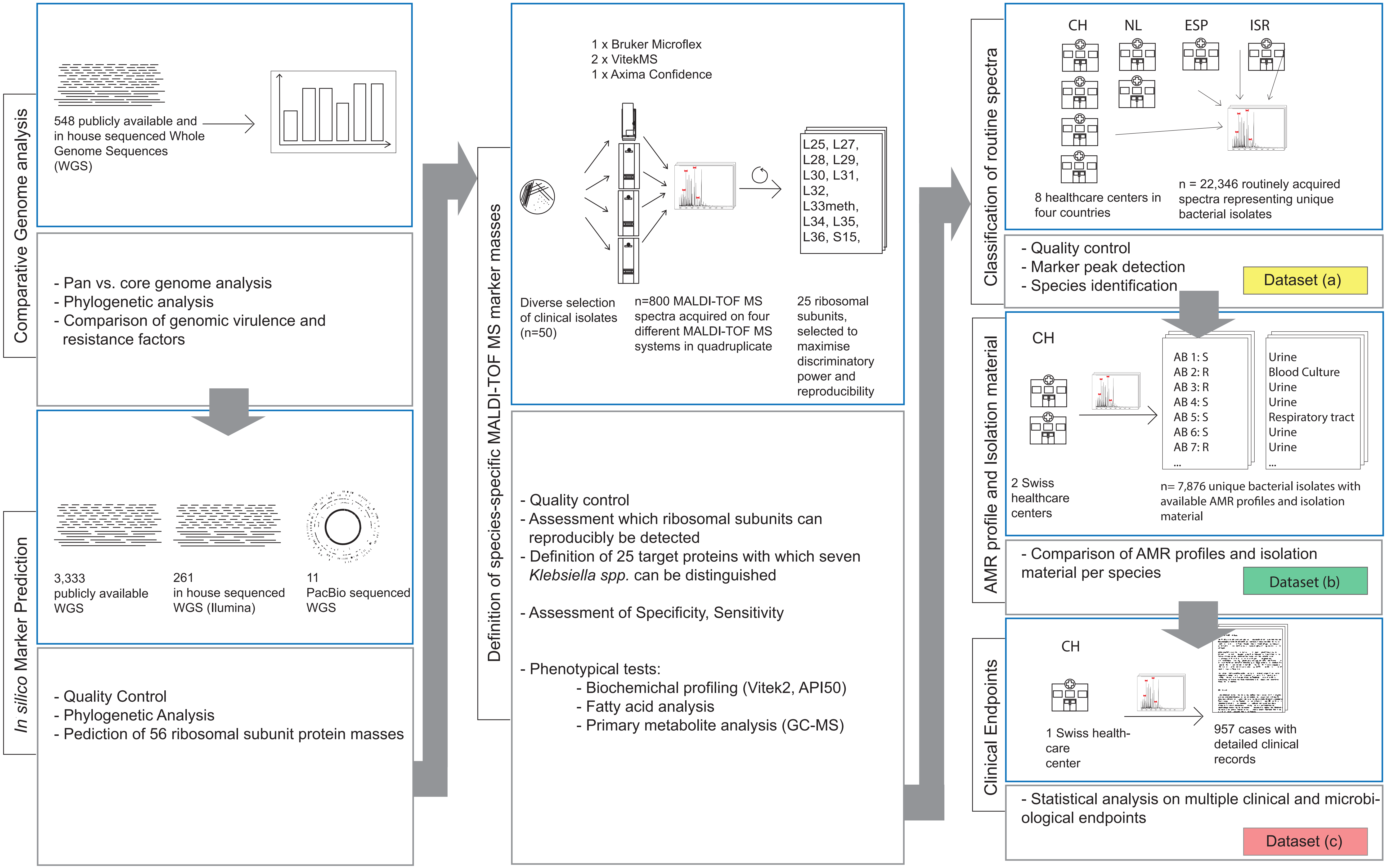

Figure S1: Workflow of the Project. Blue framed boxes indicate the data analysed whereas the grey framed boxes indicate which analysis and measurements have been done. The yellow, green and red boxes depict the datasets used in the statistical analysis comparing the occurence of *Klebsiella spp.* as well as multiple microbiological and clinical endpoints.

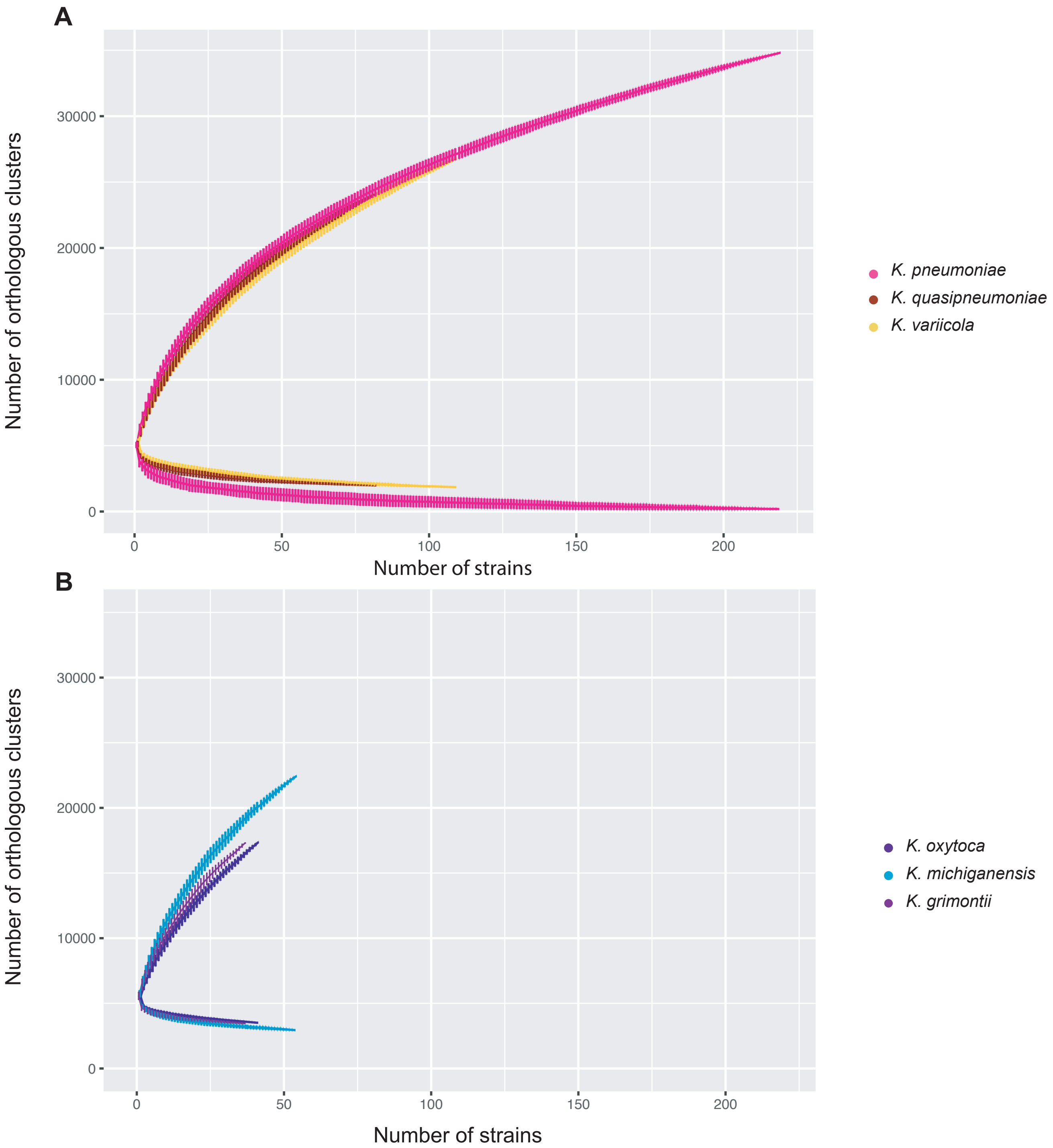

Figure S2: Gene accumulation curves for species of the *K. pneumoniae* group (**A**) and the *K. oxytoca* group (**B**)

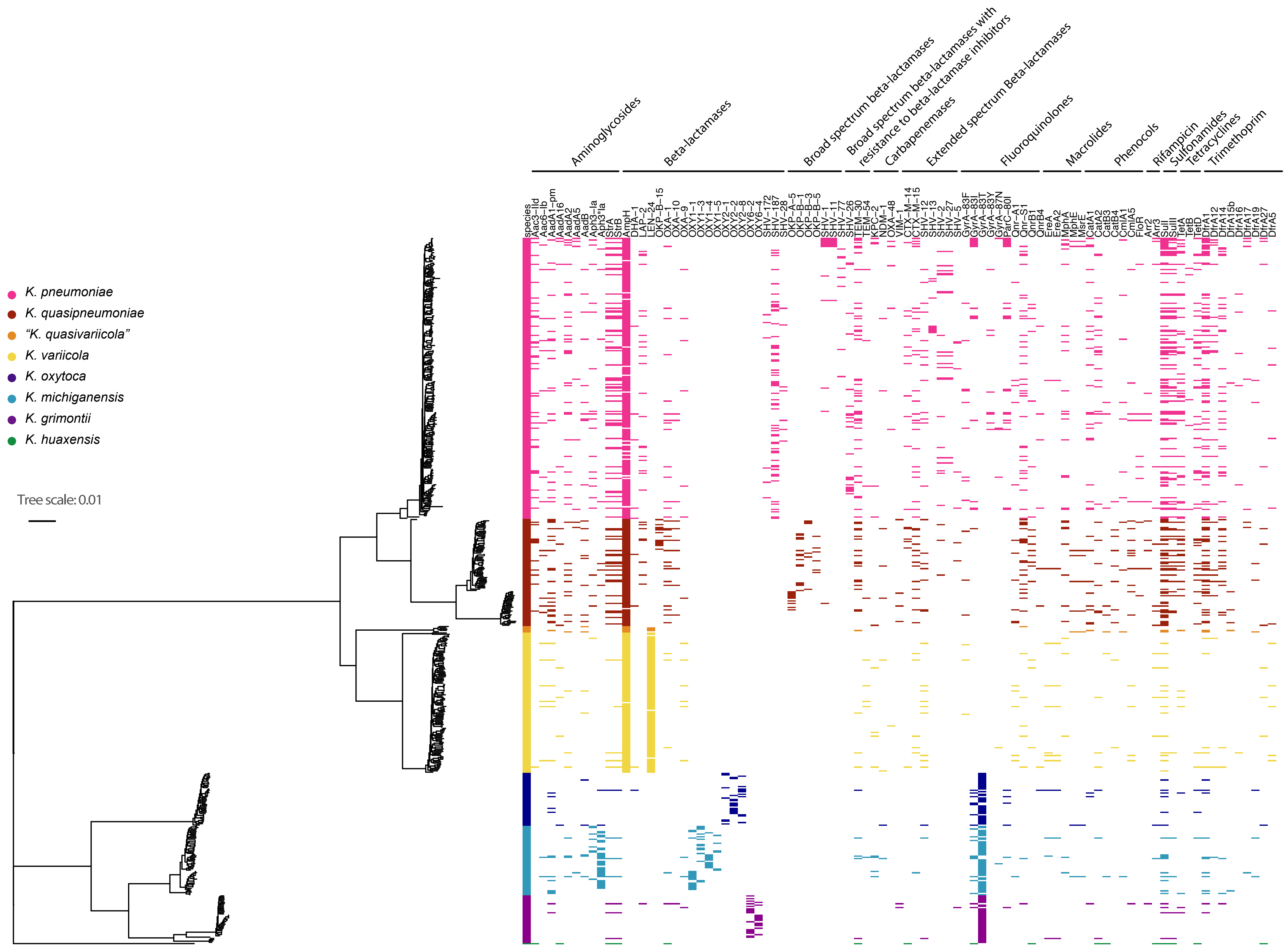

Figure S3: Genes associated with AMR detected in *Klebsiella spp.* (n=548), colored by species and grouped by antibiotic class to which they confer resistance to. Only genes which were found in six or more genomes are displayed.

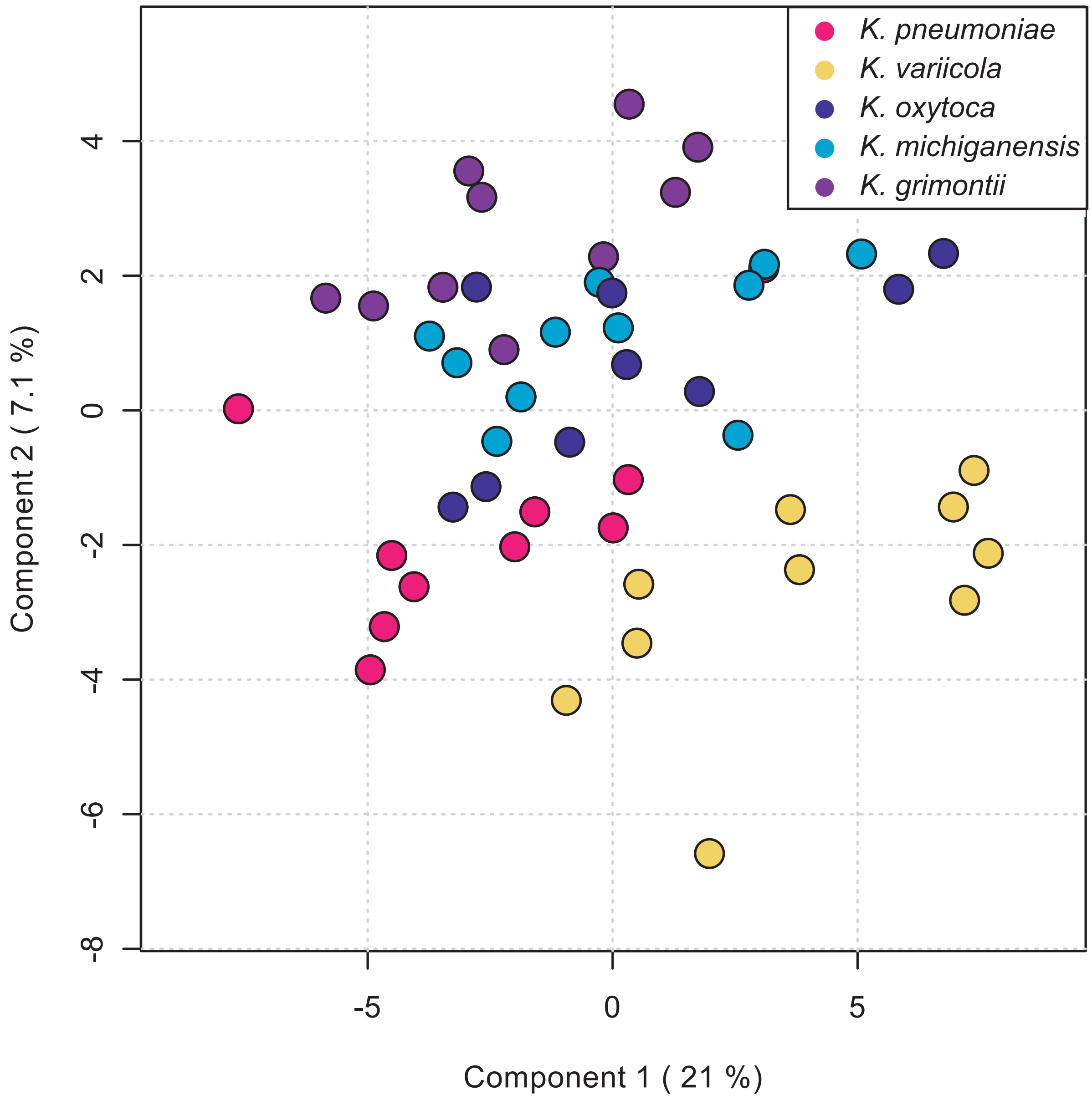

Figure S4: Partial least squares discriminant analysis (PLS-DA) score plot containing primary metabolites measured of five Klebsiella spp (n=50). The primary metabolite profile does not allow clustering of the strains according to their species identity.

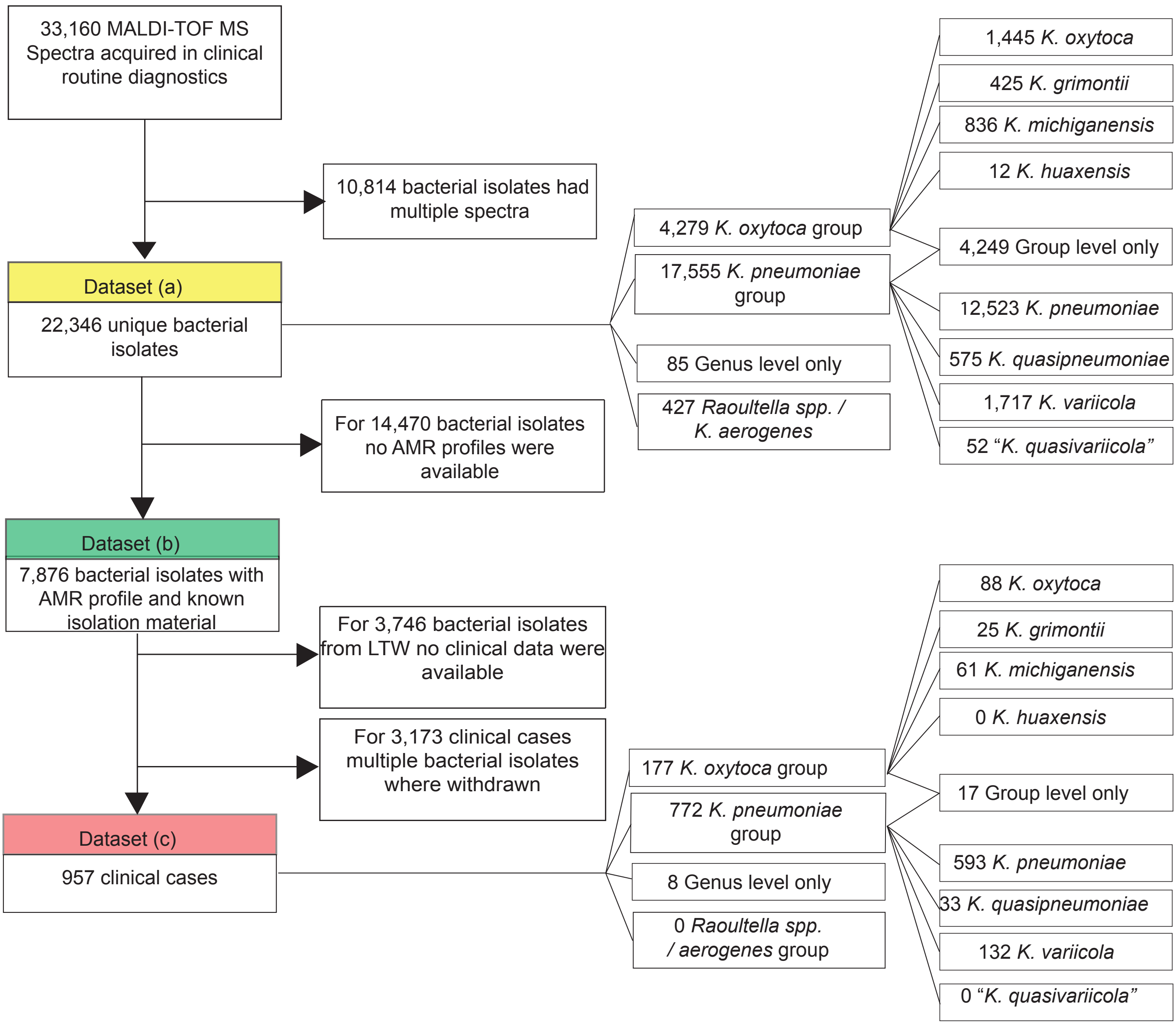

Figure S5: Species identity of datasets (a), (b) and (c) included in the statistical analysis. These three datasets were analysed with the objectives: (i) to investigate the relative distribution of *Klebsiella* spp. (and species groups) identified in samples from patients presenting with infection, with regard to center, country, sample material, and resistance to antibiotics; and (ii) to examine the link between clinical outcome of the patients and the *Klebsiella* spp. causing the infection and relevant patient characteristics.
