# Supplementary material for: Whole-genome sequence-informed MALDI-TOF MS diagnostics reveal importance of Klebsiella oxytoca group in invasive infections: a retrospective clinical study
Source: Genome Med. 2021 Sep 13;13:150. doi: 10.1186/s13073-021-00960-5 (PMC8438989; doi:10.1186/s13073-021-00960-5)
Supplement: Supplementary file 3 — Additional file 3. Supplementary methods for: Measurement of primary metabolites. Fatty acid analysis. Statistical analysis of clinical outcome data. [file 13073_2021_960_MOESM3_ESM.pdf]

Supplementary Methods

Primary Metabolites:

Sample selection

The samples included in the metabolomic analysis included a diverse selection of clinical isolates from the University Hospital Basel (n=50), representing strains of the species *K. pneumoniae*, *K. variicola*, *K. oxytoca*, *K. michiganensis* and *K. grimontii.* This is the same strain selection which has also been included in the MALDI-TOF MS marker validation and was subjected to biochemical profiling.

Sample preparation

The metabolomics experiments were performed following a procedure described by Fiehn [1]. Isolates were subcultured twice in Müller Hinton Broth media and grown until mid-exponential phase (37°C, 220 rpm). Cultures were then mixed 1:1 with ice cold 70% methanol and centrifuged for 10 min at 3 900 rcf (4°C). Cell pellets were frozen immediately in liquid nitrogen and stored at – 80°C before they were dissolved in 1000 µL ice cold (-20°C) isopropanol, acetonitrile and water mixture (3:3:2, v/v/v). The extraction solvent was degassed for 5 min under a flow of helium and cooled overnight in the freezer at -20°C before cell extraction. All samples were prepared in random order. Cells were ground with the included extraction solvent using a ball mill MM 400 (Retsch,Haan, Germany) at a vibration speed of 25 Hz for 30 seconds. The solution was further shaken at 6°C with 1400 rpm for 5 min on a thermomixer comfort (Eppendorf, Hamburg, Germany). Samples were centrifuged for 2 min at 14 000 rcf. 50 µL supernatant was evaporated to dryness with a GeneVac EZ-2 Plus (StepbioS, Muttenz, Switzerland) at 40°C. A further clean up step was done with 50 µL acetonitrile, water mixture (5:5, v/v) and centrifuged at 14 000 rcf for 2 min. The supernatant was transferred in a 500 µL Eppendorf tube and evaporated to dryness. The dry extract was re-dissolved in 10 µL in 20 mg/mL of O-methylhydroxylamine hydrochloride anhydrous pyridine solution and agitated with

1400 rpm for 90 min at 30°C. For the second derivatization step 91 µL N-methyl-N-trimethylsilyltrifluoroacetamide (MSTFA) with 1 % trimethylchlorosilan (TMCS) was added and shaken with 1400 rpm for 30 minat 37°C. The MSTFA solution 990 µL was mixed before derivatization with 10 µL of fatty acid methyl esters (FAME, C8-C16, C18-C30) for internal retention marker index.

Data acquisition

Analysis was performed on an Agilent 7890B gas chromatograph (GC) equipped with a multimode inlet. The GC was hyphenated with a quadrupole mass spectrometer 5977B (Agilent Technologies, Basel, Switzerland) equipped with an electron impact (EI) high efficiency source. Chromatographic separation was achieved on a J&W DB-5ms ultra inert column (30 m x 0.25 mm i.d. and 0.25 µm film thickness) with an integrated 10 m guard column. Helium was used as carrier gas at constant flow rate of 0.86 mL/min. An aliquot of 0.5 µL was injected into a 2 mm dimpled splitless liner at an initial temperature of 50°C that was ramped to 250°C with 12°C/second. The split vent was opened after 25 seconds. Oven temperature program started at 60°C hold for 1 min, increased to 325°C at 10°C/min and held for 10 min before cool-down. The ion source operated at 70 eV. Source temperature was set to 230°C and the transfer line was heated at 300°C. Mass spectra were acquired in scan mode between *m/z* 85 – 500 at 3.5 Hz in trace ion detection mode. The GC-MS system was controlled by MassHunter GC/MS Acquisition B.07.05 (Agilent Technologies).

Data processing

First, all raw MassHunter data files were converted to Analysis Base Files using Reifycs Abf Converter (reifycs.com/AbfConverter/). Data files with chromatographic and mass spectral data were then deconvoluted with MS Dial (v. 2.70, Sep.1, 2017) [2]. MS Dial settings were as follows; retention time start, 6.2 min; retention time end, 32 min; mass range begin, *m/z* 85; mass range end, *m/z* 500; smoothing method, linear weighted moving average; smoothing

level, 3 scans; average peak width, 20 scans; minimum peak height, 3000 amplitude; mass slice width, 0.5 Da; retention time tolerance for alignment; 0.1 min; EI similarity tolerance alignment; 70 %. Metabolite identification was done with the FiehnLib mass spectral and retention index library [3]. All aligned identified and unidentified compounds were exported to a text file. Annotated compounds were manually inspected and curated in Microsoft Excel 2016 and MassHunter Qualitative Analysis B.06.00. After curation, a reduction from 203 annotated compounds to qualified 91 compounds was achieved.

Statistical analysis

The statistical analysis was performed with MetaboAnalyst 4.0 (https://www.metaboanalyst.ca) [4,5]. A comma-separated value (.csv) file was uploaded to the server, which contains 91 compounds with peak heights. Samples are separated into five groups: *K. pneumoniae* (n= 10), *K. variicola* (n= 10), *K. oxytoca* (n= 8), *K. michiganensis* (n= 12) and *K. grimontii* (n= 10). Data containing two missing values were replaced with the half of the minimum values in the original data. For univariate analysis a one-way Analysis of Variance (ANOVA) with a p-value threshold of <0.05 was performed and adjusted using Benjamini-Hochberg false-discovery rate (FDR). Before the analysis, the values were log transformed. Group differences were determined using Tukey's Honestly Significant Difference (Tukey's HSD). For multivariate analysis, data were log transformed, sample normalisation was done by median and auto scaled. Partial least squares discriminant analysis (PLS-DA) was performed with a permutation test for model validation.

References

1. Fiehn, O. 2016. Metabolomics by gas chromatography–mass spectrometry: combined targeted and untargeted profiling. Current Protocols in Molecular Biology 114:21.33.1-21.33.11

2. Tsugawa, H., Cajka, T., Kind, T., Ma, Y., Higgins, B., Ikeda, K., Kanazawa, M., VanderGheynst, J., Fiehn, O., Arita, M. (2015). MS-DIAL: data-independent MS/MS deconvolution for comprehensive metabolome analysis. Nature methods, 12(6), 523-526.
3. Kind, T., Wohlgemuth, G., Lee, D., Y., Lu, Y., Palazoglu, M., Shahbaz, S., and Fiehn, O. 2009. FiehnLib: Mass spectral and retention index libraries for metabolomics based on quadrupole and time-of-flight gas chromatography/mass spectrometry. Analytical Chemistry 81 (24), 10038-10048
4. Xia, J. and Wishart, D.S. (2016) Using MetaboAnalyst 3.0 for Comprehensive Metabolomics Data Analysis Current Protocols in Bioinformatics, 55:14.10.1-14.10.91.
5. Chong, J., Soufan, O., Li, C., Caraus, I., Li, S., Bourque, G., Wishart, D.S. and Xia, J. (2018) MetaboAnalyst 4.0: towards more transparent and integrative metabolomics analysis. Nucl. Acids Res. 46, W486-494.

Chemicals

Isopropanol, O-methylhydroxylamine hydrochloride, anhydrous pyridine and fatty acid methyl esters were purchased from Sigma-Aldrich, Buchs, Switzerland. Acetonitrile, MSTFA + 1% TMCS silylation reagent were obtained from Fisher Scientific, Reinach, Switzerland.

Fatty acid analysis:

Sample selection

11 *Klebsiella spp.* strains were subjected to fatty acid profiling. These 11 strains included a reference strains of the species *K. pneumoniae, K. quasipneumoniae, K. variicola, K. oxytoca, K. michiganensis* one additional, clinical isolate for each species *K. pneumoniae, K. variicola, K. oxytoca, K. michiganensis*. We additionally included 2 clinical isolates of the species *K. grimontii.*

Procedure

Whole-cell fatty acids were prepared and derivatized at the stationary phase of *Klebsiella* spp. cultures grown for 24 h at 35°C on blood agar according the protocol of the Sherlock Microbial Identification System (MIDI Inc., Newark, USA). Chromatography was carried out with a Hewlett Packard (HP) 6890 gas chromatograph and data were analyzed with SHERLOCK MIS version 6.2 (MIDI Inc.)

Statistical analysis of the clinical data:

Three datasets were analysed with the objectives: (i) to investigate the relative distribution of *Klebsiella spp.* (and species groups) identified in samples from patients presenting with infection, with regard to center, country, sample material, and resistance to antibiotics; and (ii) to examine the link between clinical outcome of the patients and the *Klebsiella spp.* causing the infection and relevant patient characteristics. The three datasets, sequentially derived from each other, consisted of: dataset (a) *Klebsiella spp.* spectra acquired in clinical routine diagnostic at eight health care center from four countries that we consider to represent a unique sample (n= 22,346); dataset (b) spectra representing unique samples from the USB (Basel, Switzerland) and the LTW (Goldach, Switzerland), for which information on specimen material and antibiotic resistance pattern were available (n= 7,876); and dataset (c) cases of hospitalization in the USB, for which MALDI-TOF MS spectra, isolation material, AMR pattern and clinical outcome data were available (n=957) (**Figure S1**). In cases of infections with more than one species, the invasive *Klebsiella spp.* was considered, where present. One patient was allowed to be represented by several cases (if repeatedly hospitalized). Dataset (a) was used to elucidate the proportions of samples of each *Klebsiella spp.* and group for each center and country. Logistic regression models were performed on dataset (b), with resistance to a class of antibiotics as binary outcome, and species as single predictors. We compared the species of the *K. pneumoniae* group and the *K. oxytoca* group and the species within each

group. Since the dataset (b) includes only a few samples of *K. huaxensis* and *K. quasivariicola,* these two species were excluded from these analyses. Resistance to Aminoglycosides and Cephalosporins of the 4^{rth} generation was scarce in the species of the oxytoca group. Therefore, we omitted comparing the resistance to these two antibiotics between the species of this group.

We analysed dataset (c) in respect to the primary endpoint of ‘all-cause mortality within 30 days from *Klebsiella spp.* diagnosis’. Further, we analysed the same dataset in respect to important clinical outcomes as secondary endpoints: ‘time to death after *Klebsiella spp.* Diagnosis’, ‘whether the patient was admitted to an intensive care unit (ICU)’, ‘invasive infection to sterile sites (including the bloodstream, deep tissues, and cerebrospinal fluids)’, ‘length of hospital stays in days’, and ‘the number of medical disciplines involved to manage the specific case’, as a surrogate for case complexity. We examined the following explanatory variables for the clinical outcomes: *Klebsiella* spp. diagnosed, CCI, patient age in years at hospital entry, sex, immune suppression (defined as a dose equivalent of 20 mg prednisone / day or mentioning of immunosuppression in the patient notes), resistance to 3rd generation cephalosporins, and antibiotic treatment at the entry and during the hospital stay. For *Klebsiella spp.* as explanatory variables (binary), we compared the *Klebsiella* groups and the species within the groups.

Binary outcomes were analysed using generalized linear mixed models (GLMM) with binomial error distribution. Count outcomes (number of medical disciplines involved) were analysed using GLMM with Poisson error distribution. Time to death within hospital and length of hospital stay (time to discharge) were considered competing risks and jointly analysed by a competing risks model. The cause specific hazards were estimated using Cox proportional-hazards (PH) model. For each event of interest, the other event was used as a censoring event. Since all patients either died within the hospital or were discharged, and all dates are known, there is no other censoring (there are no patients for whom observation time ended without event). All models included all explanatory variables listed above. Since, in some

cases, more than one *Klebsiella* spp. MALDI-TOF MS spectrum could be analysed per patient, a random intercept per patient number was modeled (random effect in GLMM and frailty term in Cox PH model). Since for the binary outcomes, there was no evidence for an association with resistance to groups of antibiotics, simpler models were also fitted without resistance as explanatory variables. All statistical analysis was performed in R (v.3.4).
