# Supplementary material for: Whole-genome sequence-informed MALDI-TOF MS diagnostics reveal importance of Klebsiella oxytoca group in invasive infections: a retrospective clinical study
Source: Genome Med. 2021 Sep 13;13:150. doi: 10.1186/s13073-021-00960-5 (PMC8438989; doi:10.1186/s13073-021-00960-5)
Supplement: Supplementary file 9 — Additional file 9: Odds ratio estimates comparing Klebsiella groups and species for resistance to different antibiotic classes: Table S7. Penicillins with beta-lactamase Inhibitors. Table S8. 3rd-generation cephalosporins. Table S9. 4th-generation cephalosporins. Table S10. Aminoglycosides. [file 13073_2021_960_MOESM9_ESM.pdf]

Table S7: Odds ratio estimates for resistance to penicillins with beta-lactamase Inhibitors (PENI) by species; n=5550 samples, thereof 634 resistant to PENI.

	OR	95 % CI	p-value
K. oxytoca **group** ***vs. K. pneumoniae*** **group**	2.79	[1.70,4.63]	<0.001
K. oxytoca **vs.** ***K. michiganensis***	0.57	[0.42,0.75]	<0.001
K. oxytoca vs. K. grimontii	1.21	[0.83,1.85]	0.343
K. pneumoniae vs. K. variicola	1.8	[1.31,2.47]	<0.001
K. pneumoniae vs. K. quasipneumoniae	1.13	[0.76,1.80]	0.569

Table S8: Odds ratio estimates for resistance to 3rd generation cephalosporins (CEF3) by species; n=5567 samples, thereof 634 resistant to CEF3.

	OR	95 % CI	p-value
***K. oxytoca* group *vs. K. pneumoniae* group**	2.45	[1.31,4.58]	0.005
K. oxytoca* vs. *K. michiganensis	0.86	[0.58,1.27]	0.444
K. oxytoca vs. K. grimontii	1.28	[0.80,2.24]	0.334
K. pneumoniae vs. K. variicola	2.11	[1.42,3.18]	<0.001
K. pneumoniae vs. K. quasipneumoniae	1	[0.63,1.73]	0.988

Table S9: Odds ratio estimates for resistance to 4th generation cephalosporins (CEF4) by species; n=5567 samples, thereof 427 resistant to CEF4.

	**OR**	**95 % CI**	**p-value**
***K. oxytoca* group *vs. K. pneumoniae* group**	0.17	[0.09,0.28]	<0.001
K. pneumoniae vs. K. variicola	2.61	[1.38,5.06]	0.003
K. pneumoniae vs. K. quasipneumoniae	1.08	[0.53,2.83]	0.852

Table S10: Odds ratio estimates for resistance to aminoglycosides (AGLY) by species; n=3132 samples, thereof 213 resistant to AGLY.

	**OR**	**95 % CI**	**p-value**
***K. oxytoca* group *vs. K. pneumoniae* group**	0.22	[0.12,0.35]	<0.001
K. pneumoniae vs. K. variicola	5.8	[2.40,20.04]	<0.001
K. pneumoniae vs. K. quasipneumoniae	0.87	[0.36,2.39]	0.758
