# Supplementary material for: Whole-genome sequence-informed MALDI-TOF MS diagnostics reveal importance of Klebsiella oxytoca group in invasive infections: a retrospective clinical study
Source: Genome Med. 2021 Sep 13;13:150. doi: 10.1186/s13073-021-00960-5 (PMC8438989; doi:10.1186/s13073-021-00960-5)
Supplement: Supplementary file 10 — Additional file 10: Summary data compiled for the statistical analysis of clinical endpoints: Table S11. Summary of outcome variables for the clinical data set. Table S12. Summary of explanatory variables for the clinical data set. Statistical analyses examining differences in clinical outcome between the Klebsiella groups and species: Table S13. Odds ratio estimates from the generalized linear mixed-effects model (GLMM) for all cause death within 30 days from diagnosis. Table S14. Odds ratio estimates from the generalized linear mixed-effects model (GLMM) for ICU admission. Table S15. Hazard ratio estimates from cause-specific hazards Cox proportional hazards model for time to death within hospital after diagnosis with hospital discharge as competing event. Table S16. Estimates of the multiplicative effects from the Poisson generalized linear mixed-effects model (GLMM) for the number of medical disciplines involved. Table S17. Odds ratio estimates from the generalized linear mixed-effects model (GLMM) for the mentioning of the infection in the patient letter. [file 13073_2021_960_MOESM10_ESM.pdf]

Table S11. Summary of outcome variables for the clinical data set. The percentage of missing values is shown in the rightmost column. Time to death in hospital and hospital length of stay are competing outcomes, thus one excludes the other, leading to missing values.

	Overall	Missing
**n**	957	
**All cause death within 30 days from diagnosis (%)**	142 (14.3)	0
**All cause death in hospital (death before discharge) (%)**	86 (8.1)	0
**Time to death in hospital from diagnosis (d) (median [IQR])**	8.0 [3.0, 13.8]	85.2
**Hospital length of stay from diagnosis (d) (median [IQR])**	8.0 [4, 17.0]	14.8
**ICU admission (%)**	335 (25.9)	0
**Invasive infection (%)**	248 (25.9)	0
**Infection mentioned in letter (%)**	773 (81.1)	0.4
**No. medical disciplines involved (median [IQR])**	3.0 [2.0, 4.0]	0

Table S12: Summary of explanatory variables for the clinical data set. The percentage of missing values is shown in the rightmost column.

	Overall	Missing
**n**	957	
***Klebsiella* species (%):**		2.6
K. pneumoniae	593 (63.6)	
K. quasipneumoniae	33 (3.5)	
K. variicola	132 (14.2)	
K. oxytoca	88 (9.4)	
K. michiganensis	61 (6.5)	
K. grimontii	25 (2.7)	
**Charlson Comorbidity Index (CCI; median [IQR])**	2.0 [1.0, 3.0]	0
**Age (years) (mean (sd))**	69.9 (16.5)	0
**Female (%)**	491 (51.3)	0
**Immune suppression (%)**	188 (19.7)	0.2
**Resistance vs. Ceftriaxon (%)**	29 (3.9)	21.9
**Resistance vs. Piperaillin-Tazobactam (%)**	22 (2.9)	21.5
**Resistance vs. Carbapenem (%)**	2 (0.3)	21.5
**Antibiotic treatment at entry or during hospitalisation (%)**	841 (88.1)	0.2

Table S13: Odds ratio estimates from the generalized linear mixed-effects model (GLMM) for all cause death within 30 days from diagnosis; n = 929 complete cases with n = 139 events.

	OR	95 % CI	p-value
***K. oxytoca* group vs. *K. pneumoniae* group**	1.16	[0.42,3.23]	0.762
K. oxytoca* vs. *K. michiganensis	1.09	[0.56,2.09]	0.802
K. oxytoca* vs. *K. grimontii	1.12	[0.49,2.59]	0.798
K. pneumoniae* vs. *K. variicola	0.94	[0.55,1.60]	0.814
K. pneumoniae* vs. *K. quasipneumoniae	1.13	[0.52,2.56]	0.751
**CCI**	1.36	[1.24,1.49]	<0.001
**Age (centred; 10y-increase)**	1.16	[1.01,1.34]	0.040
**Female vs. male**	0.60	[0.40,2.18]	0.012
**Immunosuppression**	1.38	[0.87,2.18]	0.173
**Antibiotic treatment at entry or during hospitalisation**	1.39	[0.71,2.71]	0.339

Table S14: Odds ratio estimates from the generalized linear mixed-effects model (GLMM) for ICU admission; n = 732 complete cases with n = 227 events.

	OR	95 % CI	p-value
***K. oxytoca* group vs. *K. pneumoniae* group**	1.58	[0.68,3.71]	0.308
K. oxytoca* vs. *K. michiganensis	1.31	[0.71,2.40]	0.386
K. oxytoca* vs. *K. grimontii	1.49	[0.68,3.30]	0.329
K. pneumoniae* vs. *K. variicola	0.79	[0.52,1.19]	0.279
K. pneumoniae* vs. *K. quasipneumoniae	1.18	[0.64,2.19]	0.611
**CCI**	1.09	[1.00,1.18]	0.051
**Age (centred; 10y-increase)**	0.91	[0.81,1.01]	0.081
**Female vs. male**	0.63	[0.45,0.89]	0.009
**Immunosuppression**	1.22	[0.79,1.89]	0.36
**Resistance to 3rd generation cephalosporines**	1.01	[0.37,2.73]	0.98
**Antibiotic treatment at entry or during hospitalisation**	4.41	[2.10,8.93]	<0.001

Table S15: Hazard ratio estimates from cause-specific hazards Cox proportional hazards model for time to death within hospital after diagnosis with hospital discharge as competing event; n = 929 complete cases with 82 within hospital deaths and 847 discharged alive.

	HR	95 % CI	p-value
***K. oxytoca* group *vs. K. pneumoniae* group**	0.55	[0.16,1.85]	0.333
K. oxytoca* vs. *K. michiganensis	0.80	[0.35,1.87]	0.611
K. oxytoca* vs. *K. grimonitii	2.52	[0.66,9.70]	0.177
K. pneumoniae vs. K. variicola	1.00	[0.62,1.62]	0.999
K. pneumoniae vs. K. quasipneumoniae	0.78	[0.41,1.47]	0.443
**CCI**	1.13	[1.02,1.25]	0.018
**Age (centred; 10y-increase)**	1.43	[1.19,1.71]	<0.001
**Female vs. male**	0.54	[0.34,0.87]	0.011
**Immunosuppression**	1.01	[0.59,1.17]	0.983
**Antibiotic treatment at entry or during hospitalisation**	0.15	[0.41,3.21]	0.788

Table S16: Estimates of the multiplicative effects from the Poisson generalized linear mixed-effects model (GLMM) for the number of medical disciplines involved*;* n = 954 complete cases.

	Estimate	95 % CI	p-value
***K. oxytoca* group *vs. K. pneumoniae* group**	1.028	[0.834,1.269]	0.787
***K. oxytoca* vs. *K.* michiganensis**	0.996	[0.865,1.146]	0.956
K. oxytoca vs. K. grimontii	1.2	[0.999,1.443]	0.054
K. pneumoniae vs. K. variicola	1.022	[0.920,1.135]	0.67
K. pneumoniae vs. K. quasipneumoniae	0.925	[0.798,1.077]	0.287
**CCI**	1.031	[1.010,1.052]	0.003
**Age (centred; 10y-increase)**	1.019	[0.993,1.047]	0.158
**Female vs. male**	0.882	[0.812,0.959]	0.003
**Immunosuppression**	1.107	[0.998,1.226]	0.053
**Antibiotic treatment at entry or during hospitalisation**	1.252	[1.093,1.433]	0.001

Table S17: Odds ratio estimates from the generalized linear mixed-effects model (GLMM) for the mentioning of the infection in the patient letter; n = 951 complete cases with 771 events.

	**OR**	**95 % CI**	**p-value**
***K. oxytoca* group *vs. K. pneumoniae* group**	0.60	[0.21,1.63]	0.339
***K. oxytoca* vs. *K.* michiganensis**	0.82	[0.41,1.63]	0.58
K. oxytoca vs. K. grimontii	1.18	[0.52,2.72]	0.705
K. pneumoniae vs. K. variicola	1.57	[0.92,2.73]	0.115
K. pneumoniae* vs. *K. quasipneumoniae	0.66	[0.28,1.54]	0.352
**CCI**	1.04	[0.93,1.16]	0.456
**Age (centred; 10y-increase)**	1.09	[0.95,1.23]	0.21
**Female vs. male**	1.22	[0.79,1.86]	0.371
**Immunosuppression**	0.92	[0.53,1.59]	0.752
**Antibiotic treatment at entry or during hospitalisation**	56.63	[31.60,100.89]	<0.001
